# Substrate-driven assembly of a translocon for multipass membrane proteins

**DOI:** 10.1038/s41586-022-05330-8

**Published:** 2022-10-19

**Authors:** Arunkumar Sundaram, Melvin Yamsek, Frank Zhong, Yogesh Hooda, Ramanujan S. Hegde, Robert J. Keenan

**Affiliations:** 1 Department of Biochemistry and Molecular Biology, The University of Chicago, Chicago, IL USA; 2 Department of Molecular Genetics and Cell Biology, The University of Chicago, Chicago, IL USA; 3 Cell Biology Division, MRC Laboratory of Molecular Biology, Cambridge, UK

**Keywords:** Protein translocation, Endoplasmic reticulum, Chaperones

## Abstract

Most membrane proteins are synthesized on endoplasmic reticulum (ER)-bound ribosomes docked at the translocon, a heterogeneous ensemble of transmembrane factors operating on the nascent chain^1,2^. How the translocon coordinates the actions of these factors to accommodate its different substrates is not well understood. Here we define the composition, function and assembly of a translocon specialized for multipass membrane protein biogenesis^3^. This ‘multipass translocon’ is distinguished by three components that selectively bind the ribosome–Sec61 complex during multipass protein synthesis: the GET- and EMC-like (GEL), protein associated with translocon (PAT) and back of Sec61 (BOS) complexes. Analysis of insertion intermediates reveals how features of the nascent chain trigger multipass translocon assembly. Reconstitution studies demonstrate a role for multipass translocon components in protein topogenesis, and cells lacking these components show reduced multipass protein stability. These results establish the mechanism by which nascent multipass proteins selectively recruit the multipass translocon to facilitate their biogenesis. More broadly, they define the ER translocon as a dynamic assembly whose subunit composition adjusts co-translationally to accommodate the biosynthetic needs of its diverse range of substrates.

## Abstract (web summary)

Biochemical reconstitution and functional analysis reveal how newly synthesized multipass membrane proteins dynamically remodel the translocon to facilitate their successful biogenesis.

## Main

The ER translocon is built around the Sec61 complex^4^. This essential factor binds ribosomes, houses a membrane-spanning channel for polypeptide translocation, and a contains a lateral gate that opens towards the lipid bilayer for transmembrane domain (TMD) insertion^5–8^. The ER also contains members of the Oxa1 superfamily of TMD insertases^9^, including the guided entry of tail anchored protein (GET) complex^10^, the ER membrane protein complex (EMC)^11^ and TMCO1 (ref. ^12^). We recently discovered that affinity purification of TMCO1 strongly enriches for ribosome–Sec61 complexes that are translating multipass membrane proteins^3^. This ‘multipass translocon’ also contains CCDC47 and a three-protein complex comprising TMEM147, nicalin and NOMO^13^ (hereafter termed the BOS complex). Using cryogenic electron microscopy, these factors were visualized behind Sec61 (ref. ^3^), where the oligosaccharyl transferase complex (OST) ordinarily resides^14^. How the multipass translocon is recruited to this site in place of OST, why it is selective for multipass membrane proteins, and what its functions are during protein biogenesis are all poorly defined.

**Fig. 1:**
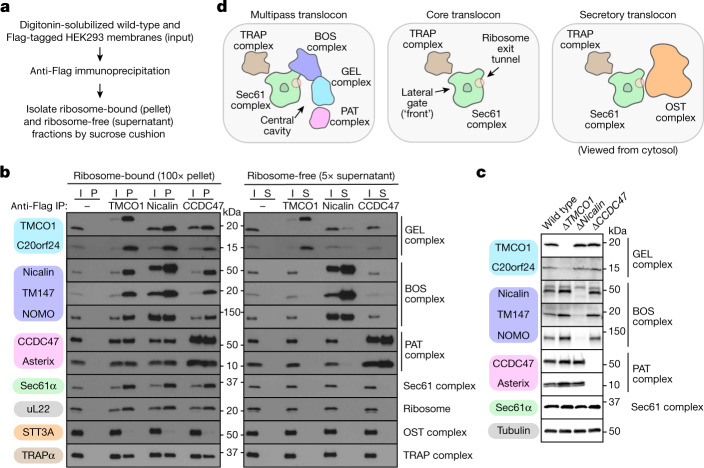
The multipass translocon is distinguished by three obligate heterocomplexes. **a**, Experimental strategy. Nuclease-treated membranes from wild-type or stably integrated Flag-tagged (*TMCO1*, *Nicalin* (also known as *NCLN*) and *CCDC47*) HEK293 cells were digitonin-solubilized, immunoprecipitated and sedimented through a sucrose cushion to isolate the ribosome-bound and ribosome-free fractions for analysis. **b**, Analysis of input (I), ribosome-bound (pellet (P)) and ribosome-free (supernatant (S)) fractions by SDS–PAGE and immunoblotting. uL22 and STT3A are used here as markers for the ribosome and OST, respectively. IP, immunoprecipitation. **c**, Whole-cell lysates from the indicated wild-type and knockout HEK293 cell lines were analysed by SDS–PAGE and immunoblotting. **d**, Subunit organization and key architectural features of the compositionally distinct multipass, core and secretory translocons, viewed from the cytosol. Source data for all gels can be found in Supplementary Fig. 1.

**Extended Data Fig. 1:**
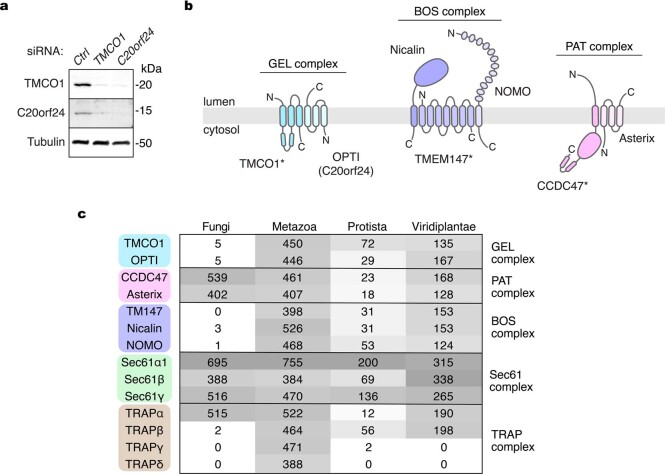
Three obligate complexes of the multipass translocon. **a**, Whole cell lysates from siRNA depleted HEK293 cells were analyzed by SDS-PAGE and immunoblotting; ‘Ctrl’ is a non-targeting control siRNA. **b**, Nomenclature, subunit topology and number of transmembrane domains for three obligate heterocomplexes of the multipass translocon. Subunits that directly contact the ribosome in the assembled state are indicated with an asterisk. Within the translocon, CCDC47 and TMEM147 contact different regions of Sec61, while interactions between the PAT, GEL and BOS complexes are limited to small portions of TMCO1 that contact CCDC47, TMEM147 and Nicalin. **c**, Distribution of the multipass translocon components in eukaryotes (OrthoDB v10.1).

As previously shown, affinity purification of epitope-tagged TMCO1 from cells co-purified ribosomes that contained the Sec61 complex, CCDC47 and the BOS complex^3^ (Fig. 1a,b). These ribosomes also contained Asterix (also known as WDR83OS), the partner of CCDC47 in the recently defined PAT complex^15,16^, and C20Orf24 (also known as RAB5IF), a recently proposed binding partner of TMCO1 (ref. ^17^). Like the PAT and BOS complex subunits, TMCO1 and C20Orf24 were mutually dependent on each other, so we named the latter OPTI (obligate partner of TMCO1 insertase; Fig. 1c and Extended Data Fig. 1a). OPTI is homologous to GET2 and EMC6, binding partners of the Oxa1 superfamily members GET1 and EMC3, respectively^17–22^. TMCO1 and OPTI are hereafter termed the GEL complex (Extended Data Fig. 1b).

TMCO1-purified ribosomes contained the TRAP complex, but did not contain OST (Fig. 1b). This is consistent with the observation that the PAT, GEL and BOS complexes occupy positions that overlap with OST, but on a different side of Sec61 to the TRAP complex^3,14^. Affinity purification using tagged subunits of the PAT or BOS complexes similarly recovered ribosomes that contain the PAT, GEL, BOS, Sec61 and TRAP complexes, but not OST. Notably, the co-purification of all of these complexes is seen only in the ribosome fraction, with little or no association observed in the ribosome-free fraction (Fig. 1b). Although the subunits within each multipass translocon complex are mutually dependent on each other, loss of any one complex does not impact the overall abundance of the others (Fig. 1c). However, because they make (limited) contact with each other at the translocon (Extended Data Fig. 1b), recruitment of each complex to the ribosome is partially dependent on the other two, as discussed later. Thus, the multipass translocon contains the PAT, GEL and BOS complexes co-assembled on ribosomes containing the Sec61 and TRAP complexes, but lacking OST (Fig. 1d). Earlier work analysing ER membranes engaged in protein secretion defined a core translocon containing only the Sec61 and TRAP complexes, and a secretory translocon that additionally contains the OST complex^14^.

**Fig. 2:**
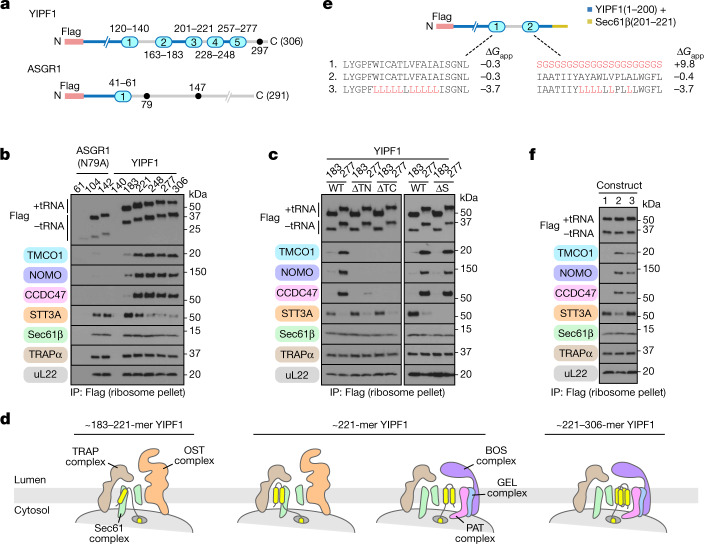
Substrate-directed assembly of the multipass translocon. **a**, Templates used to generate truncated, Flag-tagged constructs for affinity purification of stalled (no stop codon) ribosome–nascent chain complexes. All stalled intermediates are appended with Met-Leu-Lys-Val. Luminal loops (grey) and native *N*-glycosylation acceptor sites (black circles) are indicated. **b**, Stalled, Flag-tagged ASGR1(N79A) and YIPF1 constructs truncated at the indicated positions were translated in rabbit reticulocyte lysate (RRL) in the presence of wild-type HEK293 rough microsomes, and the membrane-associated fraction was isolated by sedimentation. Following anti-Flag immunoprecipitation of the digitonin-solubilized membranes, stalled ribosome–nascent chain complexes were isolated by sedimentation and analysed by SDS–PAGE and immunoblotting. Note the earliest intermediates (ASGR1 61-mer and YIPF1 140-mer) do not target to the membrane as their first TMD remains buried inside the ribosome exit tunnel, thus serving as a control for nonspecific binding. **c**, Stalled, Flag-tagged YIPF1 constructs truncated at positions 183 and 277 were translated in RRL in the presence of wild-type (WT), double-knockout (*TMCO1*/*Nicalin* (ΔTN) and *TMCO1*/*CCDC47* (ΔTC)) or *STT3A*-knockout (ΔS) rough microsomes, and analysed as in **b**. **d**, Diagram of translocon composition at different stages of YIPF1 synthesis, based on data in **b**,**c**. **e**, Series of two-TMD YIPF1 templates containing different TMD1 and TMD2 sequences. The calculated apparent free energy of membrane insertion^48^ (Δ*G*_app_) for each TMD is indicated. **f**, Stalled, Flag-tagged YIPF1 constructs as in **e** were analysed as in **b**; quantification for *n* = 5 biological replicates is shown in Extended Data Fig. 4b.

**Extended Data Fig. 2:**
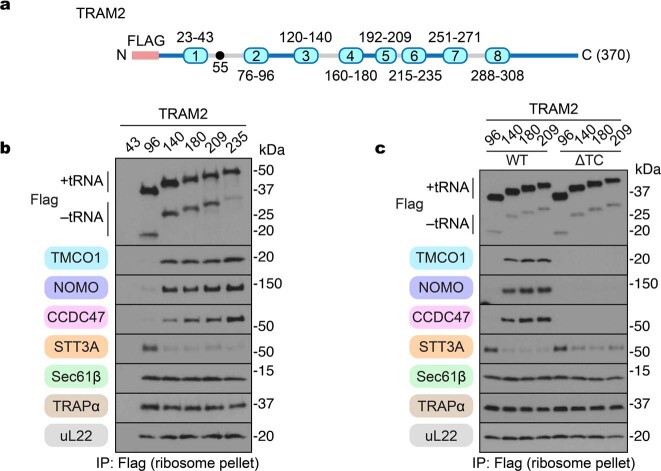
Translocon dynamics during TRAM2 synthesis. **a**, Template used to generate truncated, Flag-tagged TRAM2 constructs, as in Fig. 2a. **b**, Stalled, Flag-tagged TRAM2 constructs truncated at the indicated positions were analyzed as in Fig. 2b. Note that the 140-mer intermediate (and beyond) is glycosylated at position 55. **c**, Stalled, Flag-tagged TRAM2 constructs truncated at the indicated positions were translated in RRL in the presence of WT and double knockout (TMCO1/CCDC47, ‘ΔTC’) rough microsomes, and analyzed as in (b).

To understand the relationship between these translocons, we analysed a series of translocation intermediates assembled at ER membranes by in vitro translation (Fig. 2a). Both early and late intermediates of the single-spanning membrane protein ASGR1 were associated with the secretory translocon, but not the multipass translocon (Fig. 2b). The five-TMD protein YIPF1 (ref. ^23^), chosen because its mRNA is enriched with the affinity-purified multipass translocon^3^, behaved differently. Although early intermediates of YIPF1 contained the secretory translocon, this was markedly reduced with concomitant gain of multipass complexes at later stages (Fig. 2b). A similar result was observed using a series of intermediates of the eight-TMD protein TRAM2 (Extended Data Fig. 2a,b). The key step when this switch begins corresponds to the point when two TMDs have been membrane inserted and the third is inside the ribosome exit tunnel. Thus, OST is exchanged for the PAT, GEL and BOS complexes specifically at the point when the substrate can be minimally defined as a multipass membrane protein.

Unexpectedly, the PAT, GEL and BOS complexes were not required for substrate-triggered displacement of OST from the translocon. Even in ER membranes lacking the complexes, OST was effectively displaced by YIPF1 and TRAM2 intermediates with at least two membrane-inserted TMDs (Fig. 2c and Extended Data Fig. 2c). These results indicate that the presence of multiple TMDs in the membrane impairs OST binding to its site behind Sec61. This might be explained by a shift in the position of the inserted TMDs relative to Sec61 (ref. ^24^). Notably, the ribosome exit tunnel is offset from the central channel of Sec61 towards its back side (Fig. 1d). As a consequence, an insertion intermediate ending with a TMD whose N terminus faces the lumen (N(exo)) TMD and a 30–40-amino-acid downstream tether to the truncation point might favour the back side of Sec61 due to tension in the nascent chain (Fig. 2d). If such a TMD associates with preceding TMDs (such as TMDs linked by short loops), the presence of multiple TMDs behind Sec61 would hinder OST binding.

Consistent with this idea, an earlier structural analysis provisionally assigned an N(exo) TMD (followed by a 32-amino-acid tether) to a site behind Sec61 adjacent to OST^25^. As additional TMDs cannot be accommodated at the OST–Sec61 interface, a multipass insertion intermediate ending in this TMD–tether configuration would not be compatible with OST binding. However, the multipass complexes would be able to bind such an intermediate because there is more space between Sec61 and the multipass components^3^ (Fig. 1d). Indeed, structural and photocrosslinking analysis of a rhodopsin intermediate with three membrane-inserted TMDs in the multipass translocon revealed the third TMD in its N(exo) topology behind Sec61 and connected to the downstream tether inside the ribosome exit tunnel (see accompanying study^26^). YIPF1 and TRAM2 intermediates with two membrane-inserted TMDs are probably in the same configuration, albeit with one fewer N-terminal TMD.

**Extended Data Fig. 3:**
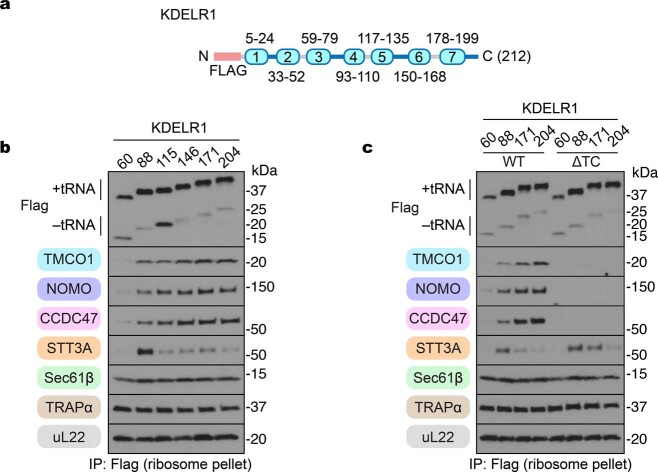
Translocon dynamics during KDELR1 synthesis. **a**, Template used to generate truncated, Flag-tagged KDELR1 constructs, as in Fig. 2a. **b**, Stalled, Flag-tagged KDELR1 constructs truncated at the indicated positions were analyzed as in Fig. 2b. **c**, Stalled, Flag-tagged KDELR1 constructs truncated at the indicated positions were translated in RRL in the presence of WT and double knockout (TMCO1/CCDC47, ‘ΔTC’) rough microsomes, and analyzed as in (b).

Similar behaviour was observed with KDELR1, a seven-TMD N(exo) protein with the opposite topology to YIPF1 and TRAM2, whose N termini face the cytosol (N(cyt)) (Extended Data Fig. 3a). The first targeted intermediate of KDELR1 engaged the core translocon (Extended Data Fig. 3b). Further elongation resulted in a mixture of secretory and multipass translocons until TMD2 and TMD3 were inserted. At this point, OST binding was reduced, with a concomitant increase in recruitment of the multipass components. OST displacement was largely independent of the multipass components (Extended Data Fig. 3c). Thus, N(cyt) and N(exo) substrates trigger displacement of OST from the translocon by a similar mechanism, except offset by one TMD.

**Extended Data Fig. 4:**
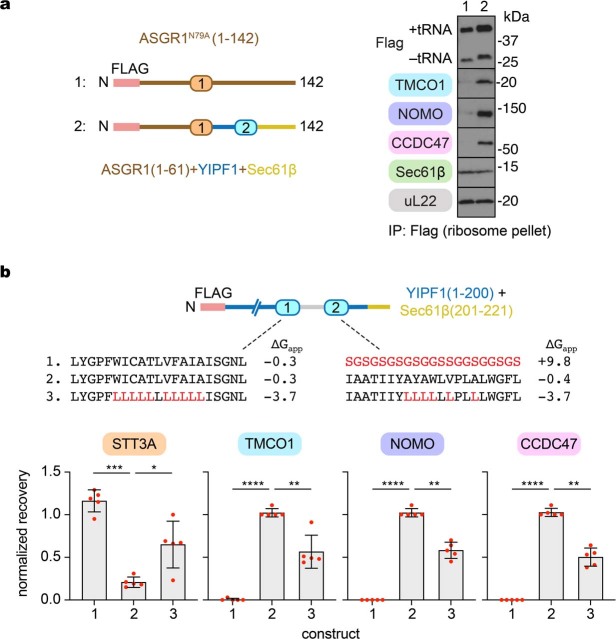
Additional characterization of substrate features that direct assembly of the multipass translocon. **a**, The indicated ASGR1-derived intermediates were translated in RRL in the presence of wild-type HEK293 rough microsomes, and the membrane-associated fraction was isolated by sedimentation. Following anti-Flag immunoprecipitation of the digitonin-solubilized membranes, stalled RNCs were isolated by sedimentation and analyzed by SDS-PAGE and immunoblotting. **b**, Quantification of STT3A, TMCO1, CCDC47 and NOMO recruitment to stalled 2-TMD YIPF1 constructs as in Fig. 2f, for n = 5 biological replicates. Data are mean ± S.D. Analysis was performed using an RM one-way ANOVA with the Geisser-Greenhouse correction and Tukey’s multiple comparisons test as implemented in GraphPad Prism 9.4.0. *, p < 0.0332; **, p < 0.0021; ***, p < 0.0002; ****, p < 0.0001; p values are given in Supplementary Table 2.

Surprisingly, displacement of OST was insufficient for assembly of the multipass translocon, because an early YIPF1 intermediate did not recruit the multipass components even in ER membranes lacking OST (Fig. 2c). To further define the trigger(s) for multipass translocon assembly, we analysed variants of the minimal recruitment intermediate containing only the first two TMDs of YIPF1 followed by a 42-amino-acid downstream tether (Fig. 2e). At this length, a mixture of secretory and multipass translocons are observed, making it a sensitive reporter of changes to this balance. The second TMD proved to be strictly required because its replacement with a hydrophilic linker abolished multipass translocon assembly (Fig. 2f). Conversely, introducing TMD2 of YIPF1 downstream of the native ASGR1 TMD was sufficient to trigger recruitment of the multipass translocon complexes (Extended Data Fig. 4a). In the two-TMD YIPF1 intermediate, increasing the hydrophobicity of both TMDs slightly (but reproducibly) reduced multipass translocon assembly in favour of secretory translocon retention (Fig. 2f and Extended Data Fig. 4b). This is consistent with the finding that TMD hydrophilicity is a key requirement for interaction with the PAT complex^15^, and perhaps other multipass translocon components. Thus, a shift in translocon composition can be triggered in two non-mutually exclusive ways: accumulation of multiple TMDs behind Sec61 to disfavour OST binding and direct TMD engagement of the multipass complexes to favour their recruitment and retention.

**Fig. 3:**
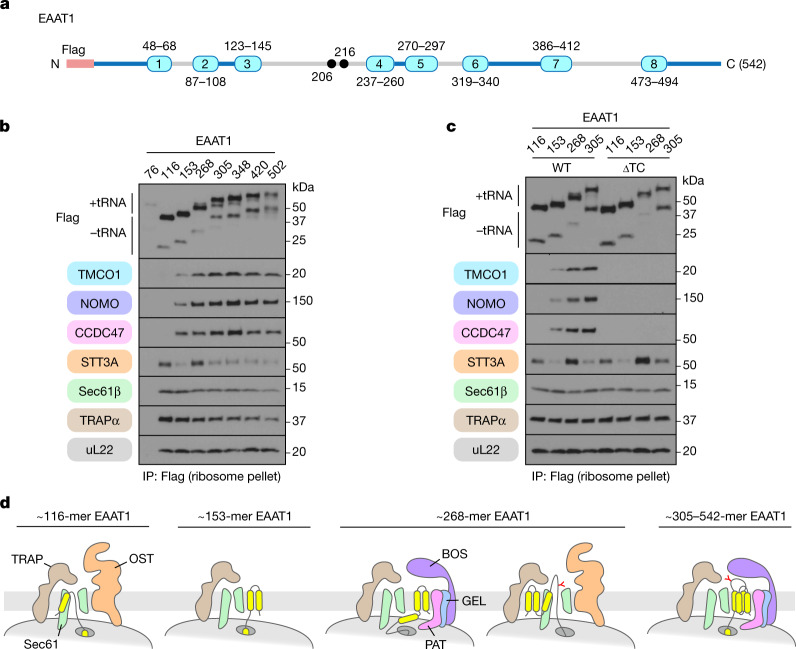
Internal loop translocation at the secretory translocon. **a**, The template used to generate truncated, Flag-tagged EAAT1 constructs, as in Fig. 2a. **b**, Stalled, Flag-tagged EAAT1 constructs truncated at the indicated positions were analysed as in Fig. 2b. The appearance of additional EAAT1 bands in later intermediates (EAAT1(268) onwards) is due to glycosylation in the TM3–TM4 luminal loop. **c**, Stalled, Flag-tagged EAAT1 constructs truncated at the indicated positions were translated in RRL in the presence of wild-type and double-knockout (*TMCO1*/*CCDC47* (ΔTC)) rough microsomes, and analysed as in **b**. **d**, Diagram of translocon composition at different stages of EAAT1 synthesis, based on data in **b**,**c**. Glycosylation of the EAAT1 acceptor site(s) is indicated.

Although most co-translationally modified glycosylation sites in multipass membrane proteins occur early, at a point when OST would still be at the translocon, at least some sites occur in long loops translocated after multiple TMDs have been inserted^27^. These loops presumably begin translocating through the Sec61 complex when the preceding TMD engages the lateral gate of Sec61 in the N(cyt) orientation. To test whether internal loop translocation occurs at the secretory translocon, we analysed biogenesis intermediates of EAAT1, an eight-TMD protein^28^ with a glycosylated luminal loop after TMD3 (Fig. 3a). As with YIPF1 and TRAM2, the earliest targeted insertion intermediate is part of the secretory translocon, after which OST is displaced when TMD2 is inserted (Fig. 3b). This EAAT1 153-mer intermediate is largely associated with the core translocon presumably because its tether length or TMD hydrophobicity limits binding to the multipass components. With elongation to a point when TMD3 has emerged from the ribosome exit tunnel, a mixture of secretory and multipass translocons are observed. Notably, the reappearance of OST at the translocon coincides with the onset of glycosylation (Fig. 3b). At later lengths OST again departs, concomitant with increased recruitment of the multipass translocon components (Fig. 3b). At each point, OST displacement occurs independently of the multipass components (Fig. 3c). We posit that TMD3 engagement of the lateral gate favours repositioning of the preceding TMDs to the front side of Sec61, allowing OST to rebind at the back side (Fig. 3d). Thus, translocon subunit composition is responsive to the nascent chain and is influenced by both the positions (relative to Sec61) and interactions of preceding TMDs.

**Fig. 4:**
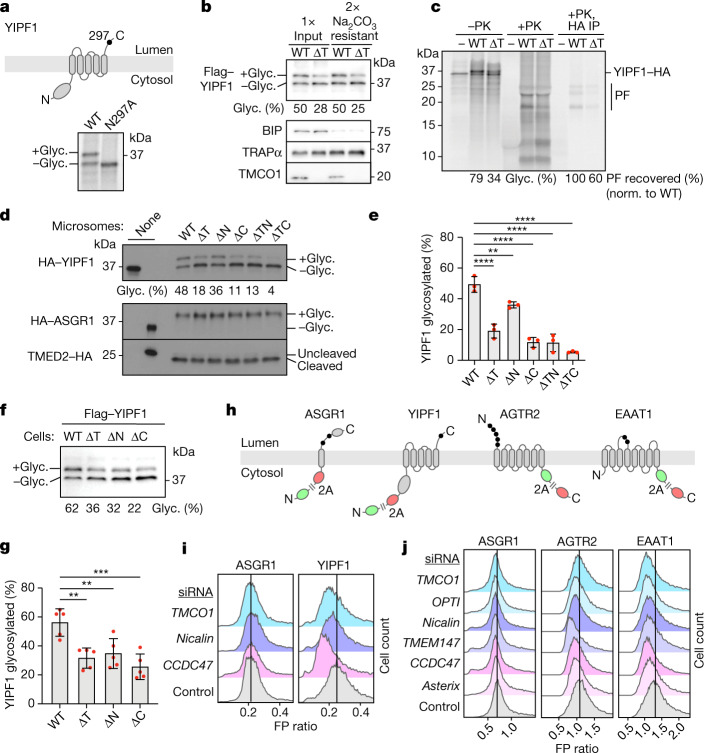
Multipass-translocon-dependent topogenesis. **a**, Top: YIPF1 harbours a single *N*-glycosylation site (black circle) near its C terminus. Bottom: [^35^S]methionine-labelled wild-type and mutant (N297A) YIPF1 were translated in RRL in the presence of rough microsomes, isolated by sedimentation, and analysed by autoradiography. **b**, Flag–YIPF1 was translated in RRL with wild-type or *TMCO1*-knockout (ΔT) rough microsomes, isolated by sedimentation, and analysed either directly (input) or after alkaline sodium carbonate extraction. YIPF1, TMCO1, BIP (ER luminal) and TRAPα (ER integral) were visualized by immunoblotting. The proportion of glycosylated (Glyc.) YIPF1 is indicated. **c**, [^35^S]methionine-labelled, C-terminally haemagglutinin (HA)-tagged YIPF1 was translated in RRL with wild-type or ΔT rough microsomes, isolated by sedimentation, and analysed by autoradiography before (−PK) or after (+PK) proteinase K treatment. The PK-treated sample was also analysed after immunoprecipitation using the HA tag. Full-length YIPF1–HA, its protease-protected fragments (PF), and the proportion of recovered PF are indicated. **d**, HA–YIPF1, HA–ASGR1 and TMED2–HA were translated in RRL with wild-type, single-knockout (*TMCO1* (ΔT), *Nicalin*, (ΔN) or *CCDC47* (ΔC)) or double-knockout (*TMCO1*/*Nicalin* (ΔTN) or *TMCO1*/*CCDC47* (ΔTC)) rough microsomes, isolated by sedimentation, and analysed by immunoblotting. **e**, Quantification of YIPF1 glycosylation for *n* = 3 biological replicates, as in **d**. The data are shown as the mean ± s.d. **f**, Flag–YIPF1 was transiently transfected into wild-type or knockout cells, and total lysates were analysed by immunoblotting. **g**, Quantification of YIPF1 glycosylation as in **f**, for *n* = 5 biological replicates. The data are shown as the mean ± s.d. **h**, Reporter constructs to monitor protein stability in cells. **i**,**j**, Stably integrated HEK293 reporter lines were treated with the indicated short interfering RNA (siRNAs), induced with doxycycline, and analysed by flow cytometry. The histograms show FP ratios for each siRNA–reporter pair; the vertical black line indicates the mode of the control population. Statistical analyses in **e**,**g** were performed by ordinary one-way analysis of variance (ANOVA) with Dunnett’s multiple comparison test (single pooled variance) in GraphPad Prism 9.4.0. **P < 0.0021; ***P < 0.0002; ****P < 0.0001; *P* values are given in Supplementary Table 1.

To analyse the consequence of multipass translocon assembly for membrane protein biogenesis, we examined the insertion of YIPF1. The single *N*-linked glycosylation site in this substrate is close to the carboxy terminus and is necessarily modified post-translationally (Fig. 4a). It therefore serves as a reporter of topogenesis errors occurring anywhere preceding it. The fraction of glycosylated YIPF1 was substantially reduced when it was inserted into Δ*TMCO1* microsomes compared to microsomes from wild-type cells (Fig. 4b). Notably, equal percentages of YIPF1 were recovered from these two reactions after carbonate extraction, indicating comparable levels of membrane insertion. As glycosylation itself is unimpaired in these microsomes (see below), the defect in YIPF1 glycosylation is likely to be a consequence of altered topology. Consistent with this idea, protease protection analysis of YIPF1 showed a reduction of around 40% of protected fragments in Δ*TMCO1* microsomes (Fig. 4c).

**Extended Data Fig. 5:**
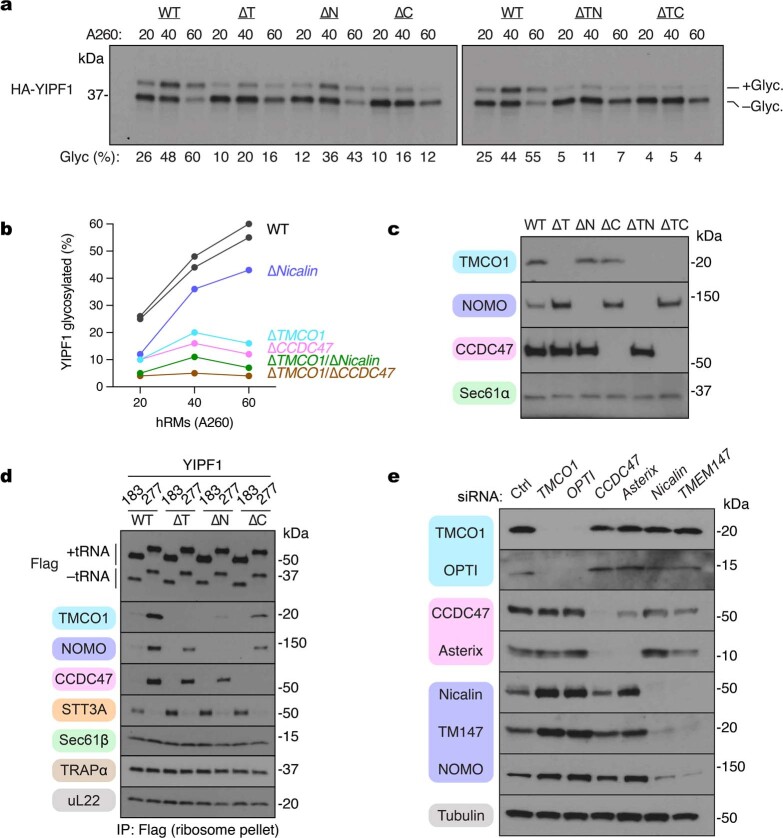
Additional characterization of the in vitro system and validation of siRNA knockdowns. **a**, HA-YIPF1 was translated in RRL in the presence of WT, single- (TMCO1, ΔT; Nicalin, ΔN; CCDC47, ΔC) or double-knockout (TMCO1/Nicalin, ΔTN; TMCO1/CCDC47, ΔTC) rough microsomes at different concentrations (determined by absorbance at 260 nm), isolated by sedimentation, and analyzed by SDS-PAGE and immunoblotting. The percentage of glycosylated YIPF1 is indicated below the gel. **b**, Plot of the data in (A). **c**, Membranes prepared from the indicated wild-type, single- or double-knockout HEK293 cell lines were analyzed by SDS-PAGE and immunoblotting. **d**, Stalled, Flag-tagged YIPF1 constructs truncated at position 183 and 277 were translated in RRL in the presence of wild-type (WT) or single knockout rough microsomes, and the membrane-associated fraction was isolated by sedimentation. Following anti-Flag immunoprecipitation of the digitonin-solubilized membranes, stalled RNCs were isolated by sedimentation and analyzed by SDS-PAGE and immunoblotting. **e**, Whole cell lysates from HEK293 cells treated with the indicated siRNAs were analyzed by SDS-PAGE and immunoblotting; ‘Ctrl’ is a non-targeting control siRNA.

Using glycosylation as a proxy for proper YIPF1 topogenesis, we observed similarly strong defects in microsomes lacking the PAT, GEL or BOS complexes, and even stronger effects in double-knockout microsomes (Fig. 4d,e). The YIPF1 defects could not be overcome by adding more microsomes to the reaction, consistent with an intrinsic biogenesis defect (Extended Data Fig. 5a,b). ASGR1 biogenesis (as judged by glycosylation) and translocation of another single-spanning membrane protein TMED2 (as judged by signal peptide cleavage) were unaffected in the same set of knockout microsomes (Fig. 4d). Thus, loss of the multipass translocon components impairs YIPF1 topogenesis without affecting SRP-dependent targeting, Sec61-mediated translocation and insertion, OST-mediated glycosylation or signal peptidase-dependent signal peptide cleavage. It is noteworthy that loss of any one multipass translocon complex reduces ribosome recruitment of the others (Extended Data Fig. 5d). For this reason, it is difficult from these data to assign the YIPF1 topogenesis defect to any one factor. Nonetheless, it is clear that the multipass translocon is functionally important for biogenesis of the multipass protein YIPF1.

Analysis of YIPF1 in cultured cells showed glycosylation defects in multipass translocon mutants similar to the in vitro results, indicating similar topogenesis defects in both systems (Fig. 4f,g). The consequence of this defect is promiscuous degradation of YIPF1 tagged with red fluorescent protein (RFP) as determined using a flow cytometry assay (Fig. 4h). In this setup, RFP–YIPF1 is translated in tandem with green fluorescent protein (GFP) but separated by the ribosome-skipping viral 2A sequence. Instability of newly made YIPF1 can be monitored as a reduction in RFP signal relative to the signal from GFP, which is necessarily translated at equal levels. The fluorescence ratio for the YIPF1 reporter was reduced in cells on knockdown of genes encoding components of the PAT, GEL or BOS complexes, consistent with impaired biogenesis (Fig. 4i and Extended Data Fig. 5e). A similar effect was seen for reporters of two unrelated multipass membrane proteins (EAAT1 and the G-protein-coupled receptor AGTR2), but not for ASGR1 (Fig. 4j). Thus, the multipass translocon is not only recruited to nascent multipass membrane proteins as monitored in vitro, but also facilitates their biogenesis in cells.

We have defined the modular composition of the multipass translocon, established its role in multipass protein topogenesis, and revealed how nascent substrates drive translocon remodelling to facilitate their successful maturation. The multipass translocon components are most broadly distributed in metazoans (Extended Data Fig. 1c). This mirrors the marked increase in membrane proteome complexity that accompanied evolution of multicellular organisms^29^. The most notable example of this expansion is seen with the seven-TMD G-protein-coupled receptors, with about nine hundred family members encoded in the human genome but only three in *Saccharomyces cerevisiae*^30^. The multipass translocon may have evolved to increase the efficiency of multipass protein biogenesis, particularly in metazoans.

Demand for multipass protein synthesis in other organisms might be satisfied by more broadly conserved components of the biogenesis machinery. In eukaryotes lacking some or all of the multipass components, the widely conserved EMC^31^ may play a more central role in multipass protein biogenesis^20,32–34^. Fungi, which lack recognizable homologues of the GEL and BOS complexes, may use their conserved PAT complex components during multipass protein synthesis at Sec61. Prokaryotes lack PAT and BOS components, but GEL complex homologues in archaea^17,35^ and the Oxa1 superfamily insertase YidC in bacteria^36–38^ may facilitate multipass protein biogenesis with SecY. In other cases, the TMD chaperone and insertase functions of the multipass components may be encoded by still unknown membrane factors.

Accommodating the diversity of secretory and membrane proteins during biogenesis requires the ER translocon to coordinate multiple transmembrane factors that operate on the nascent chain. These factors include TRAM family members^39,40^, RAMP4 (refs. ^4,41^), signal peptidase^42,43^, chaperones^44,45^, putative RNA-binding proteins^46^ and others. For multipass membrane proteins, the signals directing translocon composition proved to be multifactorial, and included negative selection (for example, by disfavouring the binding of OST on the basis of TMD number and position) and positive selection (for example, by binding to specific factors such as the PAT complex). This is analogous to the interplay between cytosolic factors at the ribosome exit tunnel during synthesis of soluble proteins^47^. Our findings provide a framework for dissecting how other biogenesis factors are coordinated at the ER translocon.

## Methods

### Antibodies and siRNAs

Antibodies to human Sec61β (1:10,000 dilution), TRAPα (ref. ^49^; 1:5,000) and TMCO1 (ref. ^9^; 1:5,000) were described previously. Other antibodies were obtained from the following commercial sources: rabbit anti-nicalin (A305-623A-M; 1:1,000) and rabbit anti-CCDC47 (A305-100A; 1:2,000) from Bethyl Laboratories; mouse anti-HA (326700; 1:1,000), goat anti-NOMO (PA5-47534; 1:1,000), rabbit anti-TMEM147 (PA5-95876; 1:1,000), rabbit anti-Asterix (PA5-66788; 1:5,000), rabbit anti-C20Orf24 (PA5-43332; 1:1,000) and rabbit anti-Sec61α (PA5-21773; 1:1,000) from Invitrogen; rabbit anti-uL22 from Abgent (AP9892b; 1:1,000); mouse anti-tubulin (ab11304; 1:1,000) and mouse anti-HRP (ab6728; 1:1,000) from Abcam; mouse anti-Flag (F1804; 1:1,000), rabbit anti-Flag (F7425; 1:1,000), rabbit anti-peroxidase (SAB3700863; 1:10,000) and goat anti-peroxidase (A5420; 1:20,000) from Sigma; mouse anti-STT3A (H00003703-M02; 1:1,000) from Novus Biologicals; mouse anti-BiP/GRP78 (610979; 1:1,000) from BD Biosciences. siRNAs were purchased from Thermo Fisher: negative control (4390843), *TMCO1* (s29085), *C20Orf24* (s31821), *Asterix* (s28089), *CCDC47* (s32576), *Nicalin* (s32411) and *TMEM147* (s20404).

### Constructs

pcDNA5 GFP–P2A–RFP–ASGR1 and pcDNA5 AGTR2–GFP–P2A–RFP fluorescent reporter constructs were described previously^32^. pcDNA5 EAAT1–GFP–P2A–RFP was constructed by Gibson cloning full-length human EAAT1 (amplified from a HEK293 cDNA library) into a modified pcDNA5/FRT/TO vector encoding a C-terminal GFP–P2A–RFP tag. pcDNA5 GFP–P2A–RFP–YIPF1 was generated by Gibson cloning a gBlock (IDT) into a modified pcDNA5/FRT/TO vector encoding an N-terminal GFP–P2A–RFP tag. Full-length constructs for in vitro translation (IVT) were generated by Gibson cloning gBlock (IDT) (YIPF1, ASGR1 and TMED2) or PCR fragments (EAAT1, KDELR1 and TRAM2) into a modified pSP64 vector encoding the desired N- or C-terminal Flag or HA tags. The 221-residue Flag–YIPF1(1–200)–Sec61β series was generated by Gibson cloning DNA fragments (Twist Biosciences) into a Flag–YIPF1 SP64 vector. The 142-residue Flag–ASGR1(1–61)–YIPF1(TM2)–Sec61β construct was generated by Gibson cloning into the parent Flag-ASGR1 SP64 vector. ASGR1(N79A) and YIPF1(N297A) substitutions were introduced using the QuikChange II Site-Directed Mutagenesis Kits (Agilent). A full-length Flag–YIPF1 construct was Gibson cloned into a pcDNA5/FRT/TO vector for in vivo glycosylation assays. All constructs were confirmed by DNA sequencing.

### Cell lines

Flp-In T-Rex 293 cells (Invitrogen) were maintained in DMEM supplemented with 10% FBS (Gemini Foundation), and 10,000 U ml^−1^ penicillin and 10 mg ml^−1^ streptomycin mixture (Invitrogen and Gemini). Cells were checked approximately every 6 months for mycoplasma contamination using the Universal Mycoplasma Detection Kit (ATCC), and were found to be negative. Single- and double-knockout (*TMCO1*, nicalin and *CCDC47*) HEK293 cell lines were generated by CRISPR–Cas9 as previously described^3,9^. An *STT3A*-knockout cell line was generated similarly. Briefly, Cas9 expression was induced by addition of 10 ng ml^−1^ doxycycline followed by transfection of single guide RNA targeting *STT3A* (5′-TCGACATTCGGAATGTCTGT-3′). Cells were grown for 48 h, followed by single-cell sorting into 96-well plates for clonal isolation. Clones were verified by both western blot and genomic sequencing. Stable cell lines expressing N-terminally Flag-tagged TMCO1 and nicalin in the corresponding knockout background were described previously^3^. A stable cell line expressing N-terminally Flag-tagged CCDC47 was generated similarly. Briefly, a *CCDC47*-knockout cell line was transfected with a modified pEGFP-n1 plasmid (Addgene) encoding N-terminally Flag-tagged CCDC47 (tag inserted after the signal peptide), under the control of a CMV promoter. Cells were transfected using TransIT-293 (Mirus) and selected in 0.7 mg ml^−1^ G418 (Invitrogen) for 2 weeks, changing the medium every 3 days. After selection, cells were maintained in medium supplemented with 0.3 mg ml^−1^ G418. Expression was verified by western blot. Stably integrated doxycycline-inducible ASGR1 and AGTR2 reporter lines for flow cytometry analysis were described previously^32^. Other reporter lines were generated similarly. Briefly, pcDNA5-based reporter constructs were co-transfected with pOG44 into Flp-In T-Rex 293 cells with TransIT-293, according to the manufacturer’s protocol (Invitrogen), and cells were selected in 100 μg ml^−1^ hygromycin B for 2 weeks.

### Preparation of rough microsomes

HEK293 cells were grown to about 80% confluency in 15-cm dishes, washed once with 5 ml ice-cold PBS (per plate) and collected by scraping in 2 × 5 ml of PBS. Cells were collected by centrifugation for 5 min at 500*g* and lysed in three volumes of hypotonic homogenization buffer (10 mM HEPES-KOH pH 7.5, 10 mM KOAc, 1 mM MgCl_2_) for 15 min on ice, with gentle agitation every few minutes. Cells were then homogenized by 15 strokes (up and down) in a chilled dounce tissue grinder. Sucrose was added to a final concentration of 250 mM and mixed gently. Nuclei and cell debris were removed by centrifugation at 700*g* for 3 min at 4 °C and the supernatant was collected. The pellet was resuspended in 5 ml of insertion buffer (50 mM HEPES-KOH pH 7.5, 250 mM sucrose, 250 mM KOAc, 10 mM MgCl_2_) and centrifuged again. The pooled supernatant fractions were centrifuged at 10,000*g* for 10 min at 4 °C. The supernatant was discarded, and the resulting membrane pellet was resuspended in insertion buffer (approximately 1 ml for about four plates). Microsomes (1-ml aliquots) were treated for 10 min at 37 °C with 4,000 U micrococcal nuclease (NEB), 2 U RNase-Free DNase (Promega), 1 mM CaCl_2_ and 0.5 mM phenylmethylsulfonyl fluoride (PMSF), followed by quenching with 2 mM EGTA. Microsomes were pelleted at 10,000*g* for 10 min at 4 °C and resuspended in 1 ml insertion buffer, 40 U SUPERaseIn and 0.1 mM EGTA, followed by centrifugation at 10,000*g* for 10 min at 4 °C. The supernatant was discarded, and the membrane pellet was resuspended in insertion buffer supplemented with 50 U SUPERaseIn (per four plates). The preparation was finally adjusted with insertion buffer to an absorbance at 260 nm (*A*_260nm_) of about 50, and 50-µl aliquots were flash frozen in liquid nitrogen and stored at −80 °C for further use.

### Interaction analysis in stably integrated cells

Microsomes from wild-type cells and cells encoding Flag-tagged versions of TMCO1, CCDC47 or Nicalin were prepared as above, except that the micrococcal nuclease digestion was performed with 10,000 U of micrococcal nuclease (NEB), 3 U DNAse (Promega), 1 mM CaCl_2_ and 0.6 mM PMSF, and incubated at room temperature for 20 min before quenching with 2.5 mM EGTA. Microsomes (1 ml at *A*_260nm_ = 50) were solubilized in insertion buffer supplemented with 2.5% digitonin and 1× protease inhibitor cocktail (Roche, 11836170001) for 45 min on ice and then diluted twice with 150 mM KOAc insertion buffer. Digitonin-solubilized microsomes were cleared by centrifugation at 12,500*g* for 15 min at 4 °C. The cleared supernatant (*A*_260nm_ of about 3.5) was immunoprecipitated in batch format using 50 µl M2 Flag affinity beads (Sigma, A2220) and gentle agitation overnight at 4 °C. Flow-through was removed and beads were washed three times with eight column volumes of insertion buffer supplemented with 0.4% digitonin. Bound material was eluted twice, for 30 min on ice, with two column volumes of 200 mM KOAc insertion buffer supplemented with 0.5 mg ml^−1^ Flag peptide (ApexBio, A6001) and 0.4% digitonin. The eluate was collected using a pre-equilibrated spin filter column (Thermo Fisher, 69725). Ribosome-free and ribosome-bound fractions were obtained by pelleting the eluate through a 300-µl sucrose cushion (50 mM HEPES pH 7.4, 10 mM MgCl_2_, 150 mM KCl, 500 mM sucrose and 0.4% digitonin) at 355,000*g* for 1 h at 4 °C in a TLA120.1 rotor.

### In vitro transcription and translation

In vitro transcription reactions utilized PCR-based templates containing an SP6 promoter, and were carried out at 40 °C for 1 h (ref. ^50^). Unless otherwise noted, reactions contained 5–10 ng μl^−1^ PCR product, 40 mM HEPES pH 7.6, 6 mM MgCl_2_, 2 mM spermidine, 10 mM dithiothreitol, 500 μM ATP, 500 μM UTP, 500 μM CTP, 100 μM GTP, 0.5 mM m7G(5′)ppp(5′)G RNA Cap, 0.4 U μl^−1^ SUPERaseIn and 0.4 U μl^−1^ SP6 RNA Polymerase. IVT reactions were performed using a RRL system (Green Hectares). Translation reactions contained 20% (v/v) of the unpurified transcription reaction, 33% (v/v) haemin- and micrococcal nuclease-treated RRL, 0.2 μCi μl^−1^ [^35^S]methionine (or 40 μM methionine for non-radioactive IVT reactions), 0.1 mg ml^−1^ bovine liver transfer RNA, 13 mM HEPES, 10 mM creatine phosphate, 1 mM ATP, 1 mM GTP, 9 mM KOH, 25 mM KOAc, 1 mM MgCl_2_, 40 μM of the remaining 19 amino acids and 10% (v/v) HEK293-derived microsomes (typically *A*_260nm_ about 50). Translation reactions were carried out for 45 min at 32 °C, unless otherwise noted.

### Interaction analysis of stalled ribosome–nascent chain complexes in vitro

Templates for the synthesis of stalled N-terminally Flag-tagged substrates were PCR amplified using reverse primers encoding a terminal Met-Leu-Lys-Val (5′-CACCTTGAGCAT-3′) sequence and lacking a stop codon. In vitro transcription and translation reactions were performed essentially as described above. Briefly, 100 µl of in vitro transcription mix containing 500 ng of purified PCR template was incubated for 1 h at 40 °C. Translation reactions of 500 µl contained 60 µl microsomes (*A*_260nm_ of about 50) and 100 μl of the unpurified transcription reaction, and were carried out for 50 min at 32 °C. The translation reactions were stopped by diluting them with 500 µl IVT stop buffer, and microsomes were pelleted at 12,500*g* for 10 min. Microsomes were washed again with 1 ml IVT stop buffer, centrifuged at 12,500*g* for 10 min, and resuspended with 500 μl IVT stop buffer. The resuspension was then treated with 5,000 U micrococcal nuclease (NEB), 1 mM CaCl_2_ and 0.6 mM PMSF at room temperature for 20 min. The reaction was stopped with 2.5 mM EGTA, and centrifuged at 12,500*g* for 10 min. The resulting membrane pellet was solubilized with 200 μl insertion buffer supplemented with 2.5% digitonin and 1× Protease Inhibitor for 45 min on ice, then diluted 2× with 150 mM KOAc insertion buffer, and cleared by centrifugation at 12,500*g* for 15 min. The cleared supernatant (*A*_260nm_ of about 1.0) was incubated overnight at 4 °C with 20 µl M2 Flag affinity beads. Flow-through was removed and beads were washed 3 times with 18 column volumes of insertion buffer containing 0.4% digitonin and 200 mM KOAc. Bound material was eluted twice with two column volumes of 200 mM KOAc insertion buffer supplemented with 0.5 mg ml^−1^ Flag peptide and 0.4% digitonin, by incubating for 30 min each on ice. The eluate was collected using a pre-equilibrated spin filter column. Eluted material was layered over a 300-µl sucrose cushion (50 mM HEPES pH 7.4, 10 mM MgCl_2_, 150 mM KCl, 500 mM sucrose and 0.4% digitonin) and pelleted at 355,000*g* for 1 h at 4 °C in a TLA120.1 rotor. The ribosomal pellet was resuspended in a 35-µl sucrose cushion buffer, normalized by *A*_260nm_, and then analysed by SDS–PAGE and immunoblotting.

### Carbonate extraction

IVT reactions (150 µl) synthesizing Flag–YIPF1 were diluted tenfold with IVT stop buffer and membranes were centrifuged for 10 min at 10,000*g*. The membrane pellets were resuspended with 150 μl IVT stop buffer, and one-third of the reaction was reserved as the input fraction. The remaining material was incubated with 100 volumes of 100 mM Na_2_CO_3_ (pH 11.5) for 30 min on ice. The sample was centrifuged at 214,000*g* for 40 min in a TLA100.3 rotor to isolate membranes, and the supernatant was discarded. This was repeated once to remove contaminating proteins. The resulting carbonate-extracted membranes were resuspended with 1× LDS sample buffer for analysis.

### Protease protection assays

YIPF1–HA was synthesized in three 150-μl IVT reactions either lacking or containing microsomes. Immediately following synthesis, the samples were diluted with ten volumes of IVT stop buffer. Microsome-containing samples were centrifuged at 10,000*g* for 10 min to pellet membranes and remove haemoglobin, and then resuspended to 150 µl with IVT stop buffer. All three samples were then split into three equal fractions for PK analysis. The untreated fractions (−PK) were set aside, and PK was added to the other samples (+PK) to a final concentration of 0.5 mg ml^−1^ and incubated on ice for 45 min. The digestion was quenched with 5 mM PMSF and incubated on ice for 5 min, followed by addition of ten volumes of boiling 1% SDS, 100 mM Tris pH 8 and 1× Roche cOmplete Protease inhibitor cocktail. For analysis of the total PK-treated fraction, samples containing microsomes were TCA-precipitated to concentrate membranes before SDS–PAGE analysis. For HA immunoprecipitations, samples were diluted tenfold with immunoprecipitation buffer (1× PBS, 250 mM NaCl, 0.5% (v/v) Triton X-100) and 30 μl HA agarose resin (Pierce, 26181) was added. Samples were incubated for 2 h at 4 °C with gentle agitation, washed three times with 1 ml of immunoprecipitation buffer, and eluted by adding 50 µl 1× LDS sample buffer and incubating at 70 °C for 10 min.

### Glycosylation analysis in vitro

IVT reactions (35 µl) synthesizing HA–YIPF1, HA–ASGR1 or TMED2–HA were carried out for 50 min at 32 °C. Immediately following translation, microsomes were washed twice with 15 volumes of IVT stop buffer (50 mM HEPES pH 7.5, 200 mM NaCl, 10 mM MgCl_2_) and then collected by centrifugation at 13,000*g* for 10 min. Membrane pellets were lysed with 35 μl IVT stop buffer containing 1.5% DDM for 30 min on ice, and centrifuged at 13,000*g* for 10 min. A 30 µl volume of lysed material was diluted with 75 µl of 3× LDS sample buffer containing 2% β-ME, heated at 65 °C for 10 min, and then analysed by SDS–PAGE and immunoblotting. Reactions without microsomes (30 µl) were supplemented with 1.5% DDM, diluted with 100 µl of 3× LDS sample buffer containing 2% β-ME, and loaded at one-tenth the amount relative to the microsome samples.

### Glycosylation analysis in cells

At 24 h before transfection, wild-type, Δ*TMCO1*, Δnicalin and Δ*CCDC47* HEK293 cells were seeded at 400,000 cells per well onto a poly-lysine-coated 6-well plate, in triplicate. A transfection mixture containing 1 μg pcDNA5 Flag–YIPF1, 150 μl Opti-MEM and 3 μl TransIT-293 was incubated at room temperature for 25 min before being added dropwise to each well. A final concentration of 1 ng ml^−1^ doxycycline was added to induce Flag–YIPF1. Following 12 h of induction, cells were collected by scraping with chilled 1× PBS. Cells were pelleted at 500*g* for 5 min and resuspended in 100 μl RIPA buffer (50 mM Tris pH 7.4, 150 mM NaCl, 1% NP-40, 0.5% sodium deoxycholate, 0.1% SDS, 1 mM PMSF, 1× Roche cOmplete Protease Inhibitor Cocktail). RIPA lysis samples were incubated on ice for 15 min and gently vortexed every 5 min. The samples were centrifuged at 15,000*g* for 10 min, and the supernatant was collected for SDS–PAGE and western blot analysis.

### Flow cytometry analysis of reporter cell lines

The effect of different siRNAs on stably expressed reporter cell lines was analysed using flow cytometry as described previously^15^. siRNA depletion was performed over a period of about 72 h using the Lipofectamine RNAiMAX reagent (Thermo Fisher) according to the manufacturer’s instructions. Briefly, a first round of siRNA treatment was performed in the presence of DMEM and 10% tetracycline-free FCS. Cells were incubated for 48 h, and then a second round of siRNA treatment was performed. After a second incubation of about 24 h, expression of fluorescent reporter constructs was induced with 1,000 ng ml^−1^ doxycycline for 6 h before analysis by flow cytometry. In all experiments the cells were collected by trypsinization, washed once in ice-cold PBS, and then resuspended 1 ml of PBS. Cells were passed through a 70-μm filter before flow cytometry analysis using a Becton Dickinson LSRII instrument. Live cells were gated by forward and side scatter. Additional gating for relatively high levels of the soluble fluorescent protein reporter was used to focus on the population of cells with productive translation of reporter constructs. Between 15,000 and 30,000 GFP-positive (EAAT1 and AGTR2) or RFP-positive (ASGR1 and YIPF1) cells were collected. Data were analysed using FlowJo (version 10.8).

### Statistics and reproducibility

Biochemical experiments in vitro and functional assays in cells were repeated in part or in full on separate and independent occasions with similar results. Fully replicated experiments include each of the following (with the number of repeats in parentheses): Figs. 2f (*n* = 5), 3b (*n* = 2) and 4a–d,f,i,j (*n* = 5, 3, 5, 3 (for YIPF1) 5, 2 and 2, respectively) and Extended Data Figs. 1a (*n* = 2), 3b (*n* = 2), 4a (*n* = 3) and 5c (*n* = 2). Other experiments were partially replicated in pilot experiments not shown, or as part of other experiments. These include: Figs. 1b,c, 2b,c, 3c and 4d (ASGR1 and TMED2) and Extended Data Figs. 2b,c, 3c and 5a,d,e. For example, the interaction analysis in Fig. 2b was performed once as shown, but it was first piloted by monitoring recruitment of a subset of components by several key ASGR1 and YIPF1 intermediates; in addition, the YIPF1 intermediate series was repeated once in its entirety with identical results. In other instances, parts of one experiment were validated by replication in another experiment (for example, Fig. 2b,c and Extended Data Figs. 2b,c and 3b,c). Therefore, these experiments can be considered to have been reproduced at least once, even when the experiment shown was not formally repeated. Statistical analysis of replicates of the data shown in Fig. 4d,f (*n* = 3 and 5, respectively) was performed by ordinary one-way ANOVA with Dunnett’s multiple comparison test (single pooled variance) in GraphPad Prism 9.4.0. Statistical analysis of replicates of the data shown in Fig. 2f (*n* = 5) was performed by repeated measures one-way ANOVA with the Geisser–Greenhouse correction and Tukey’s multiple comparison test. The bar graphs in Fig. 4e,g and Extended Data Fig. 4b show the individual data points, mean and s.d. *P < 0.0332; **P < 0.0021; ***P < 0.0002; ****P < 0.0001. The source data can be found in Supplementary Tables 1 and 2.

### Reporting summary

Further information on research design is available in the Nature Research Reporting Summary linked to this article.

## Online content

Any methods, additional references, Nature Research reporting summaries, source data, extended data, supplementary information, acknowledgements, peer review information; details of author contributions and competing interests; and statements of data and code availability are available at 10.1038/s41586-022-05330-8.

## Supplementary information

Supplementary Information This file contains Supplementary Figs. 1 and 2.

Reporting Summary

Supplementary Table 1

Supplementary Table 2

## Data Availability

Data generated in this study are available within the article and Supplementary Information. Source data for all gels can be found in Supplementary Fig. 1. The gating strategy for flow cytometry experiments can be found in Supplementary Fig. 2. Source data for the graphs shown in Fig. 4e,f can be found in Supplementary Table 1. Source data for the graphs shown in Extended Data Fig. 4b can be found in Supplementary Table 2.
