# Supplementary material for: Substrate-driven assembly of a translocon for multipass membrane proteins
Source: Nature. 2022 Oct 19;611(7934):167–72. doi: 10.1038/s41586-022-05330-8 (PMC9630114; doi:10.1038/s41586-022-05330-8)

nature portfolio

https://doi.org/10.1038/s41586-022-05330-8

Supplementary information

Substrate-driven assembly of a translocon for multipass membrane proteins

In the format provided by the authors and unedited

Supplementary Figure 1 | Uncropped western blots and autoradiographs.

Figure 1b – left panel

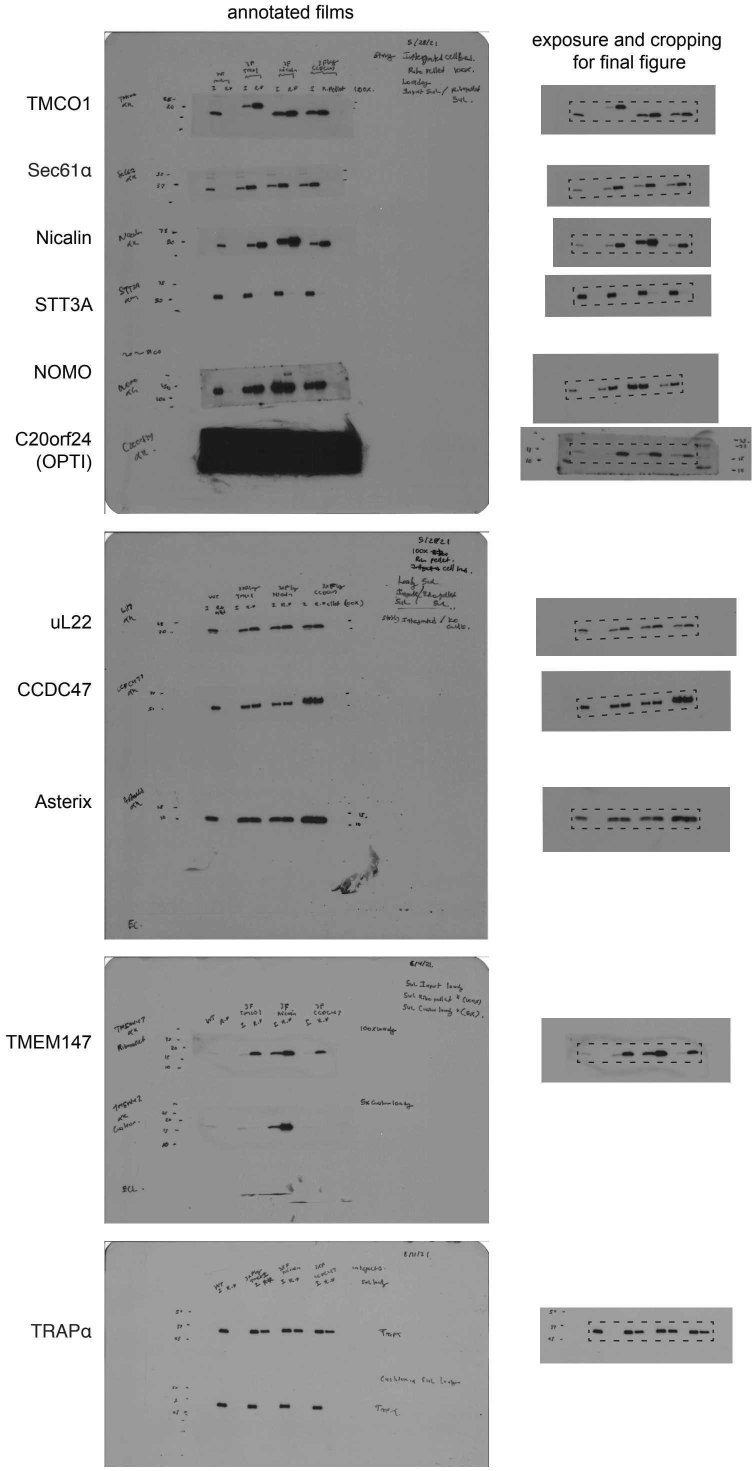

Figure 1b – right panel

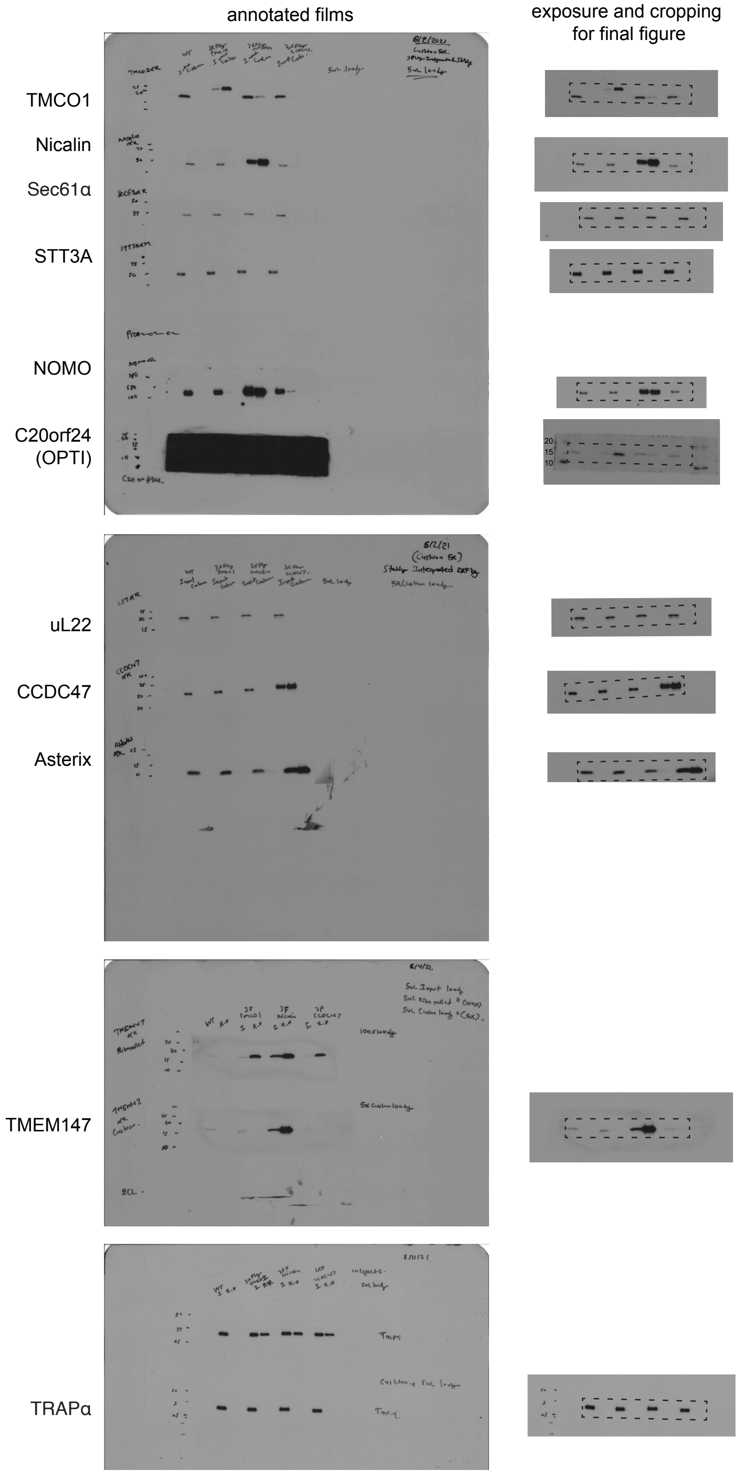

Figure 1c

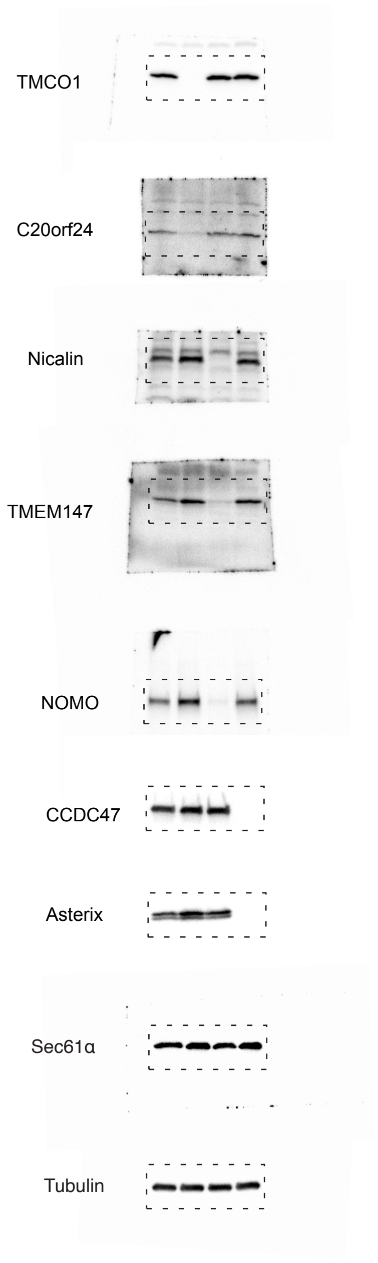
TMCO1
C20orf24
Nicalin
TMEM147
NOMO
CCDC47
Asterix
Sec61α
Tubulin

Figure 2b

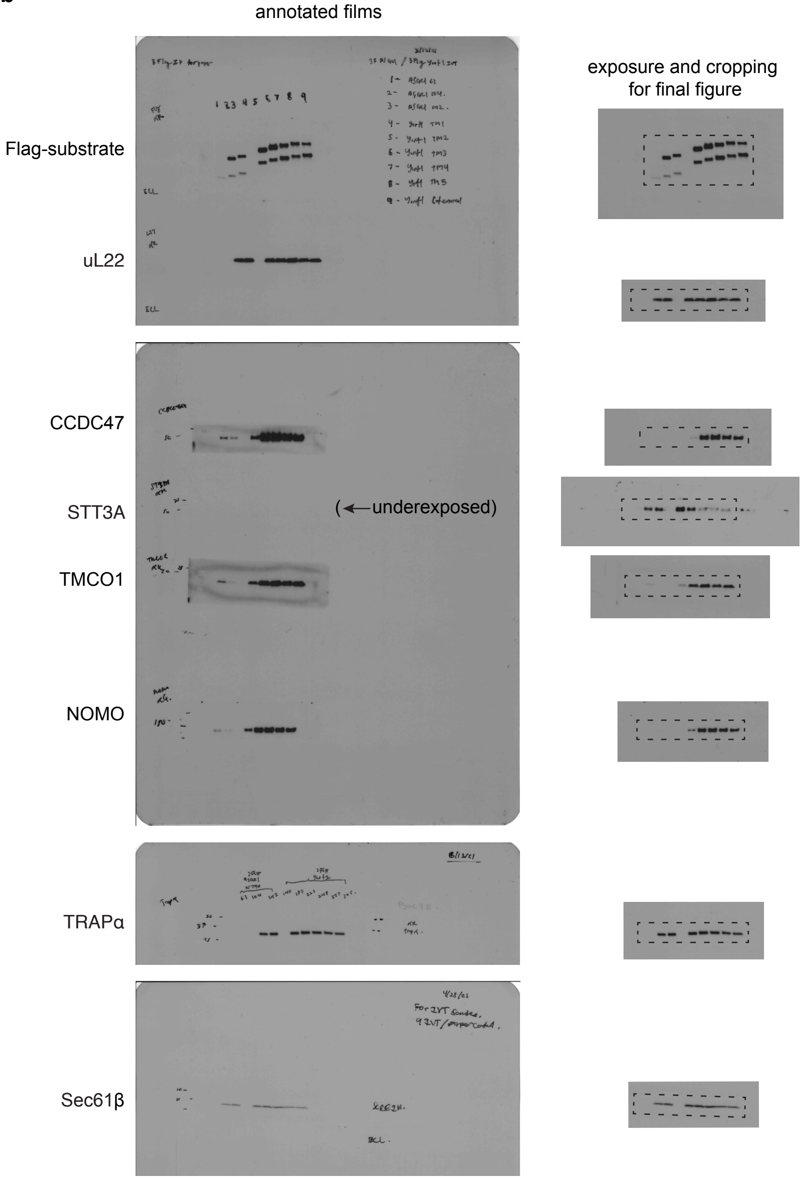
annotated films
exposure and cropping
for final figure
Flag-substrate
uL22
CCDC47
STT3A
(←underexposed)
TMCO1
NOMO
TRAPα
Sec61β

Figure 2c

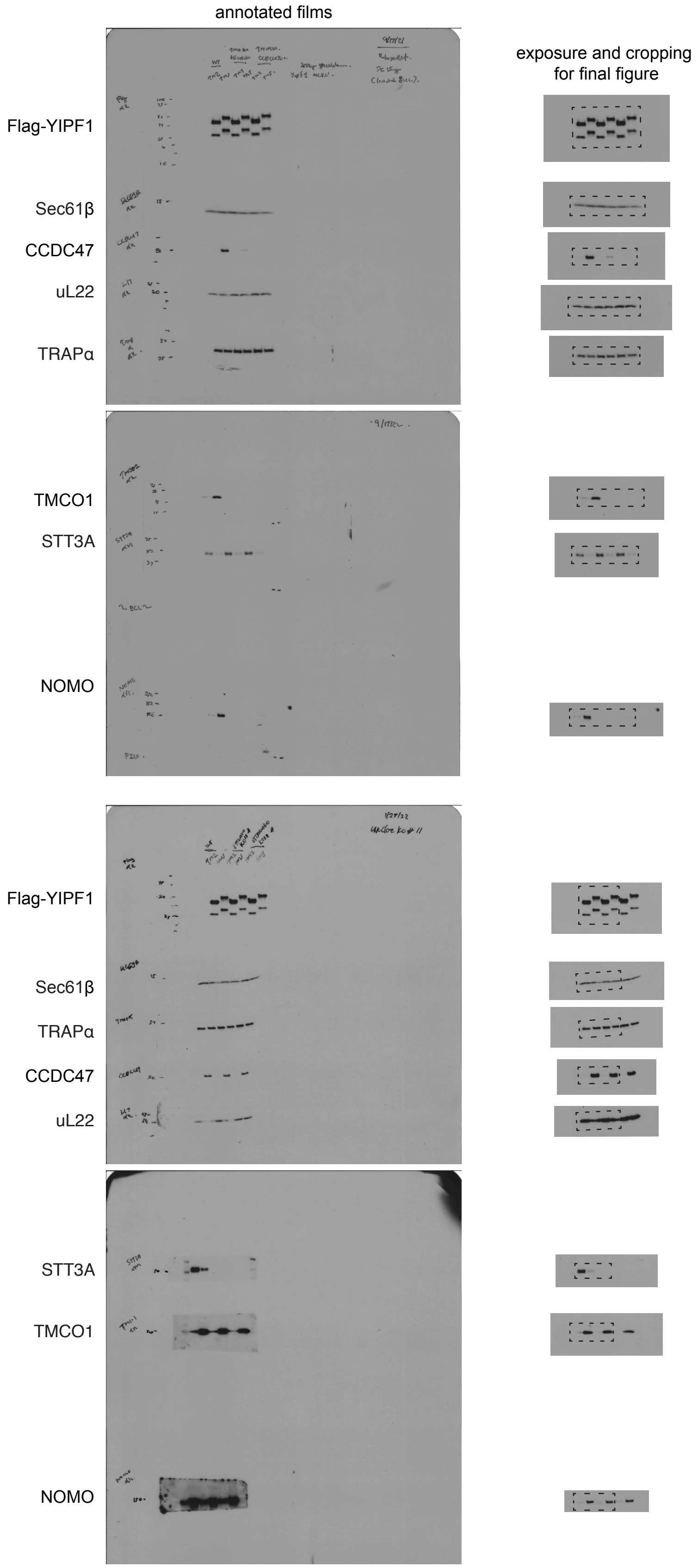
annotated films
exposure and cropping for final figure
Flag-YIPF1
Sec61β
CCDC47
uL22
TRAPα
TMCO1
STT3A
NOMO
Flag-YIPF1
Sec61β
TRAPα
CCDC47
uL22
STT3A
TMCO1
NOMO

Figure 2f

annotated films

exposure and cropping for final figure

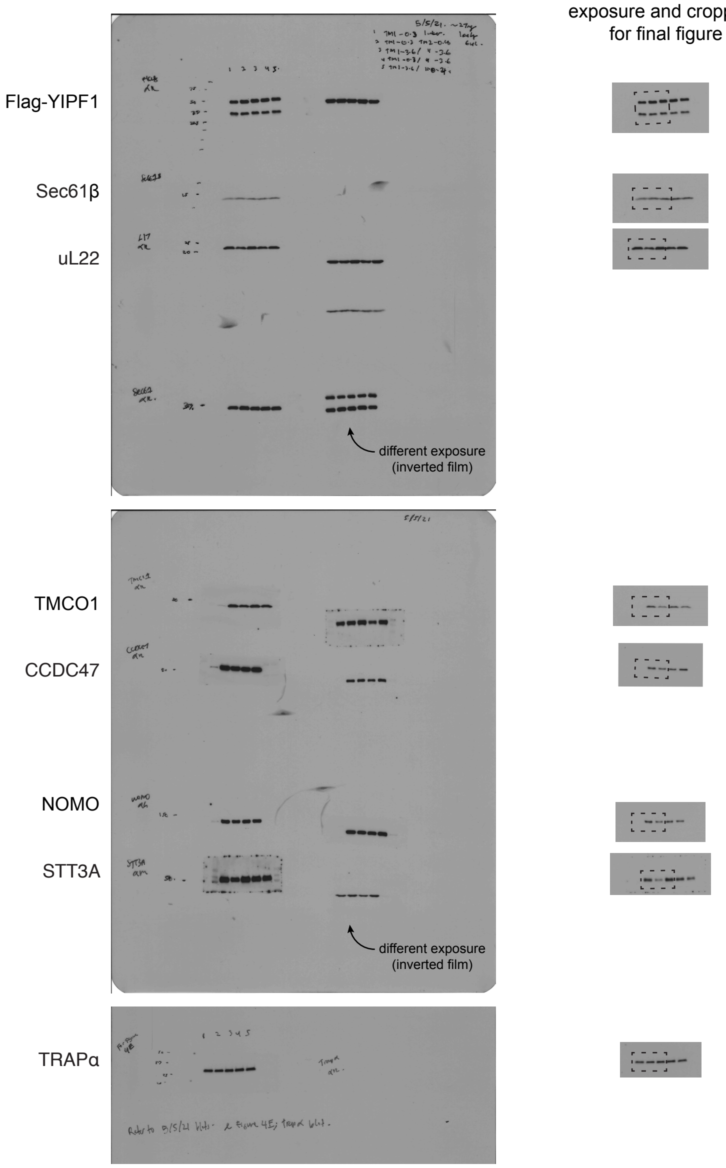

Figure 3b

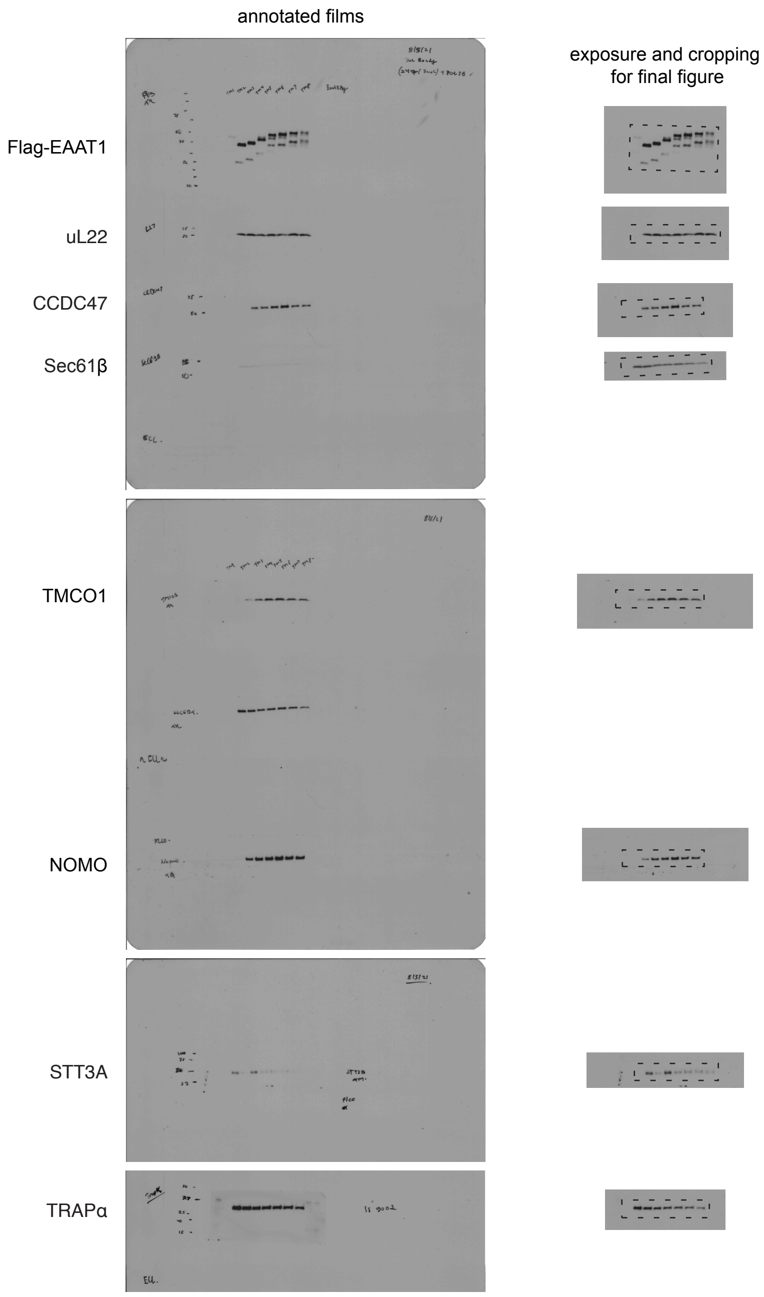
annotated films
exposure and cropping for final figure
Flag-EAAT1
uL22
CCDC47
Sec61β
TMCO1
NOMO
STT3A
TRAPα

Figure 3c

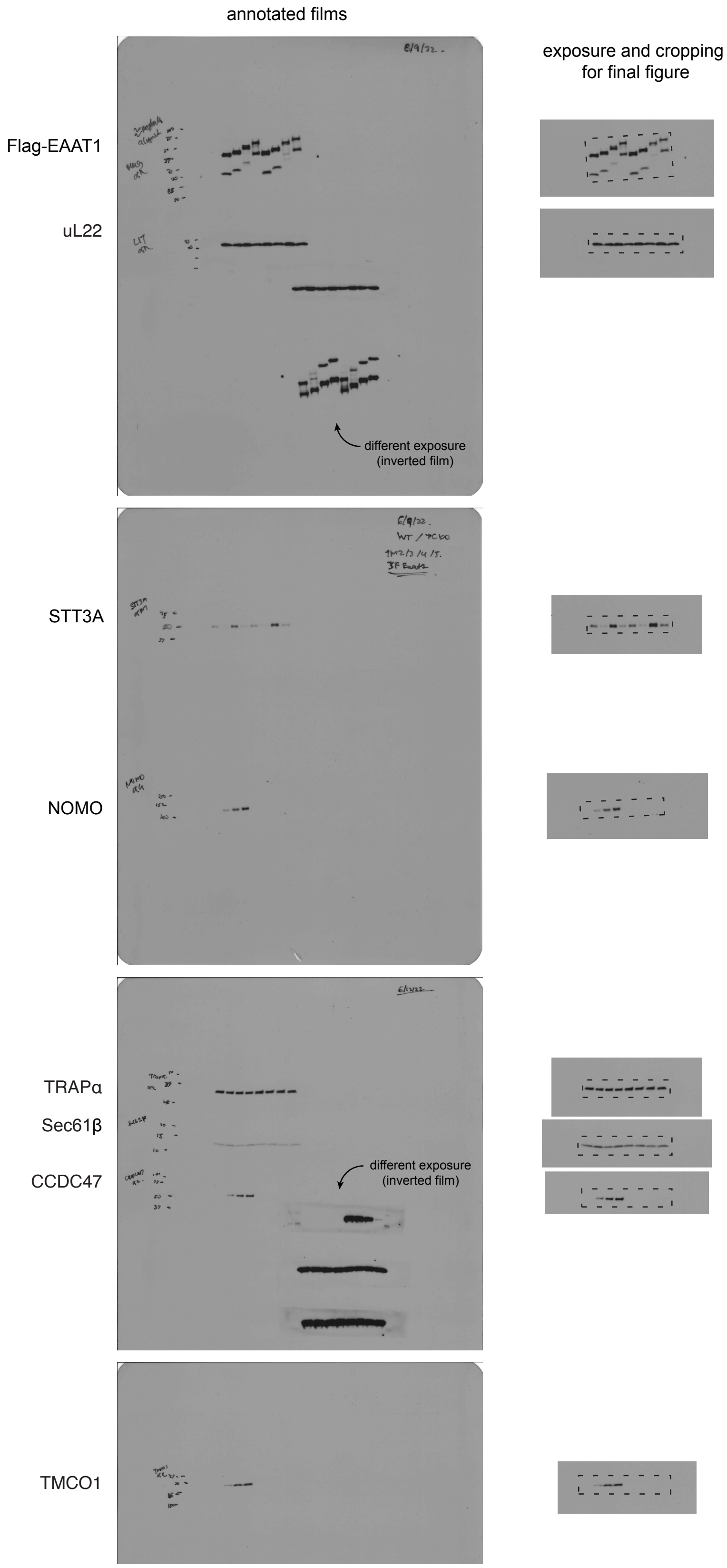
annotated films
exposure and cropping for final figure
Flag-EAAT1
uL22
different exposure (inverted film)
STT3A
NOMO
TRAPα
Sec61β
CCDC47
different exposure (inverted film)
TMCO1

Figure 4

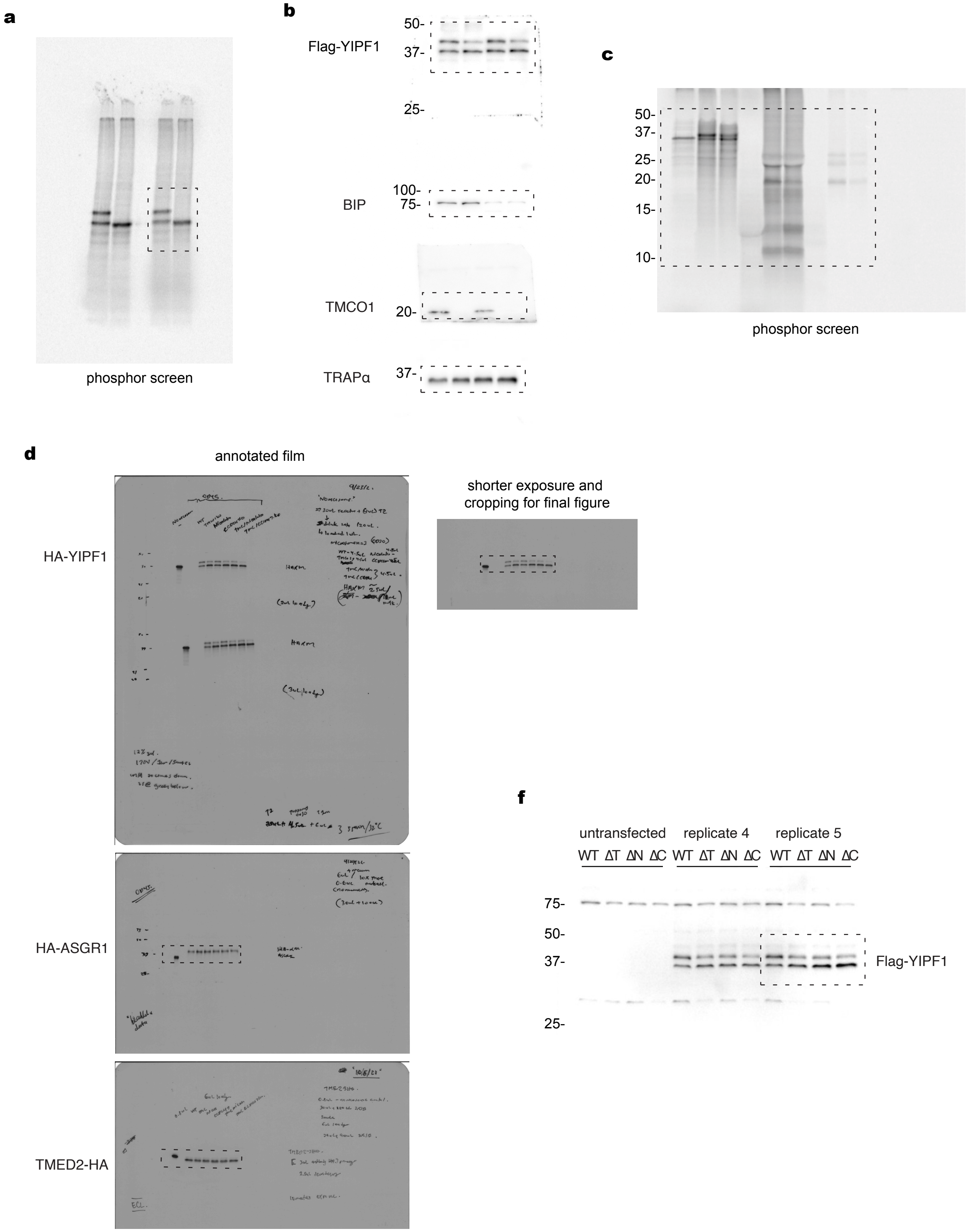
a
phosphor screen
b
Flag-YIPF1
50-
37-
25-
BIP
100-
75-
TMCO1
20-
TRAPα
37-
c
50-
37-
25-
20-
15-
10-
phosphor screen
d
annotated film
HA-YIPF1
HA-ASGR1
TMED2-HA
shorter exposure and cropping for final figure
f
untransfected
replicate 4
replicate 5
WT ΔT ΔN ΔC WT ΔT ΔN ΔC WT ΔT ΔN ΔC
75-
50-
37-
25-
Flag-YIPF1

Extended Data Figure 1a

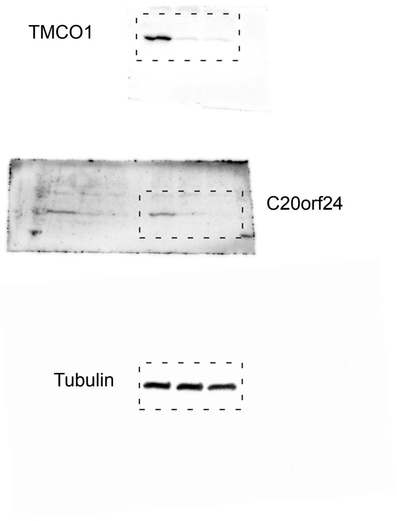

Extended Data Figure 2b

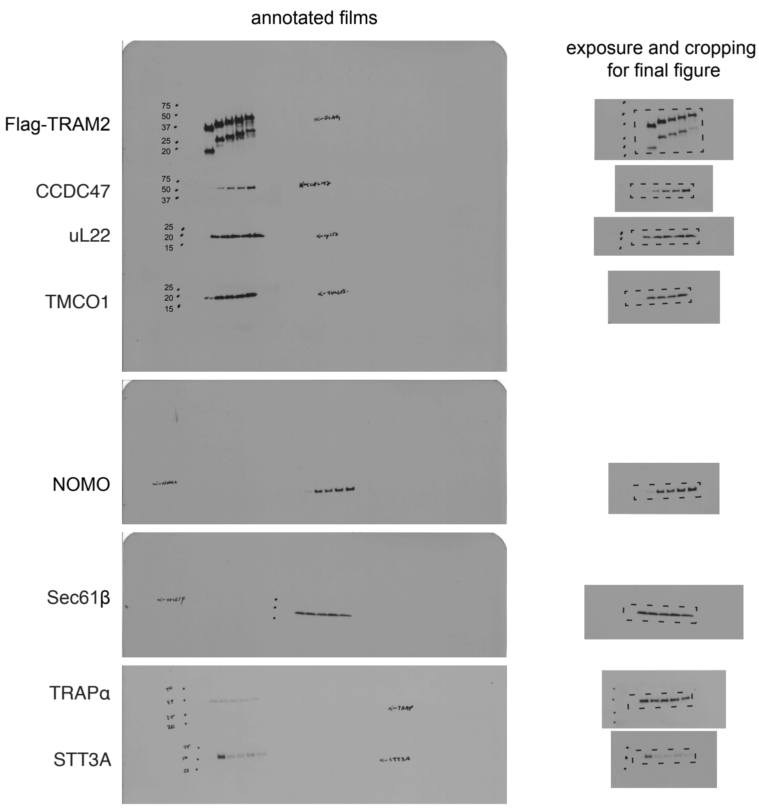

Extended Data Figure 2c

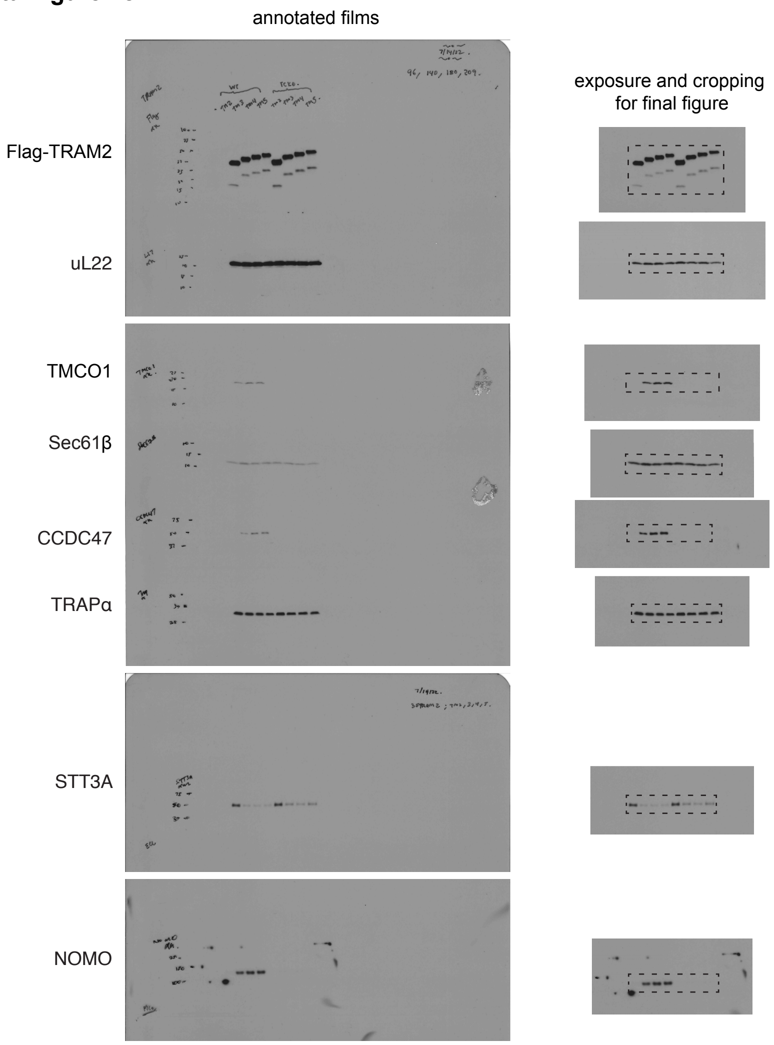

Extended Data Figure 3b

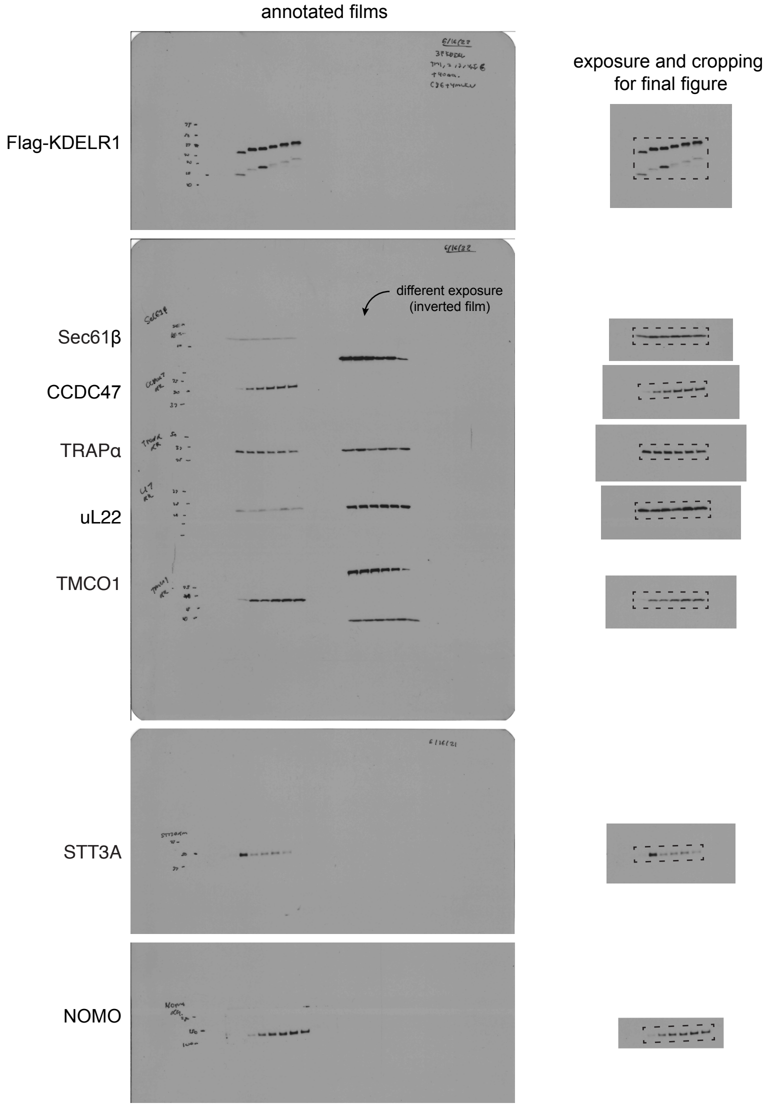

Extended Data Figure 3c

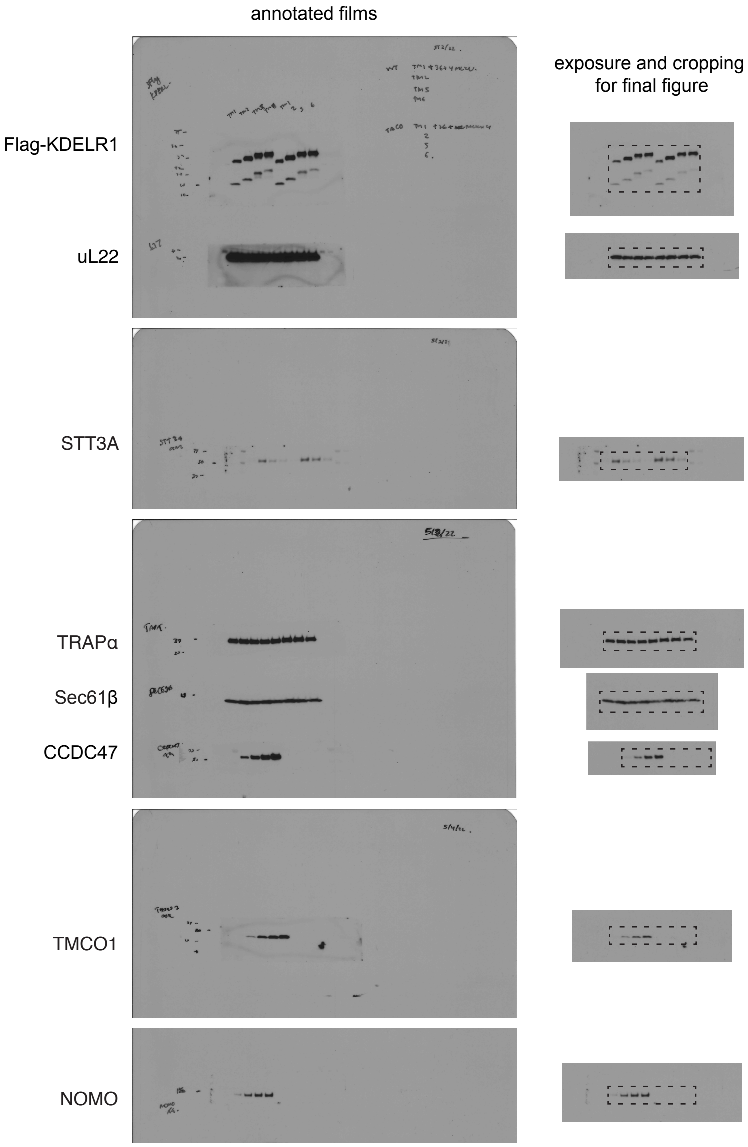

Extended Data Figure 4a

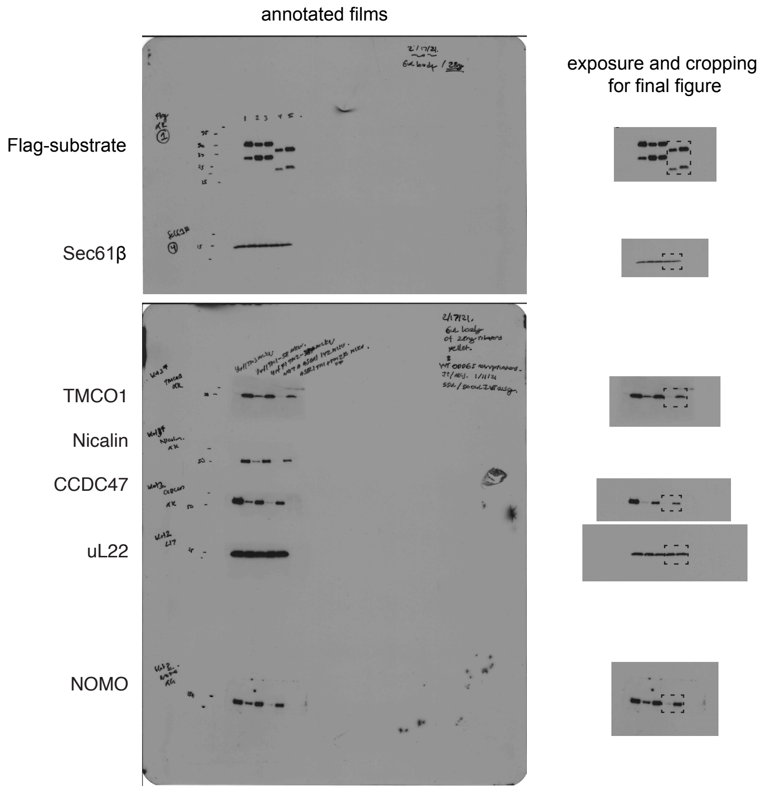

Extended Data Figure 5a,c

a

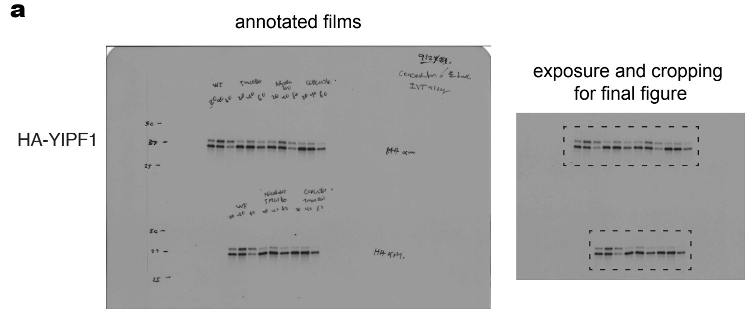

c

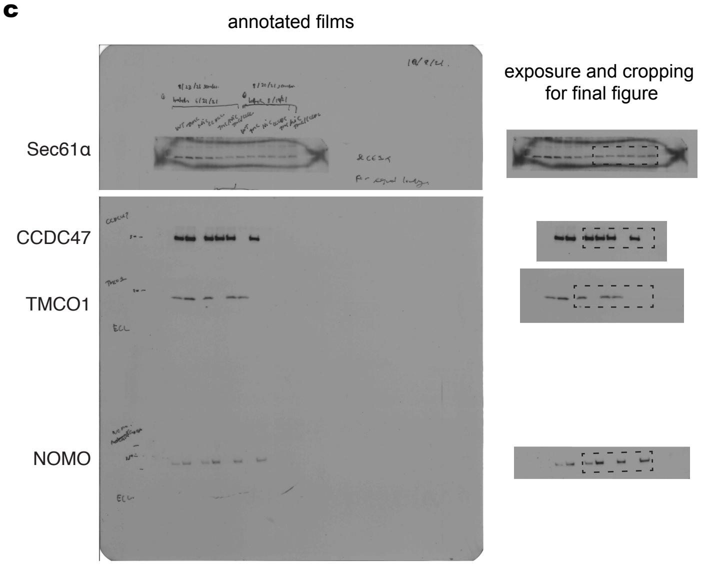

Extended Data Figure 5d

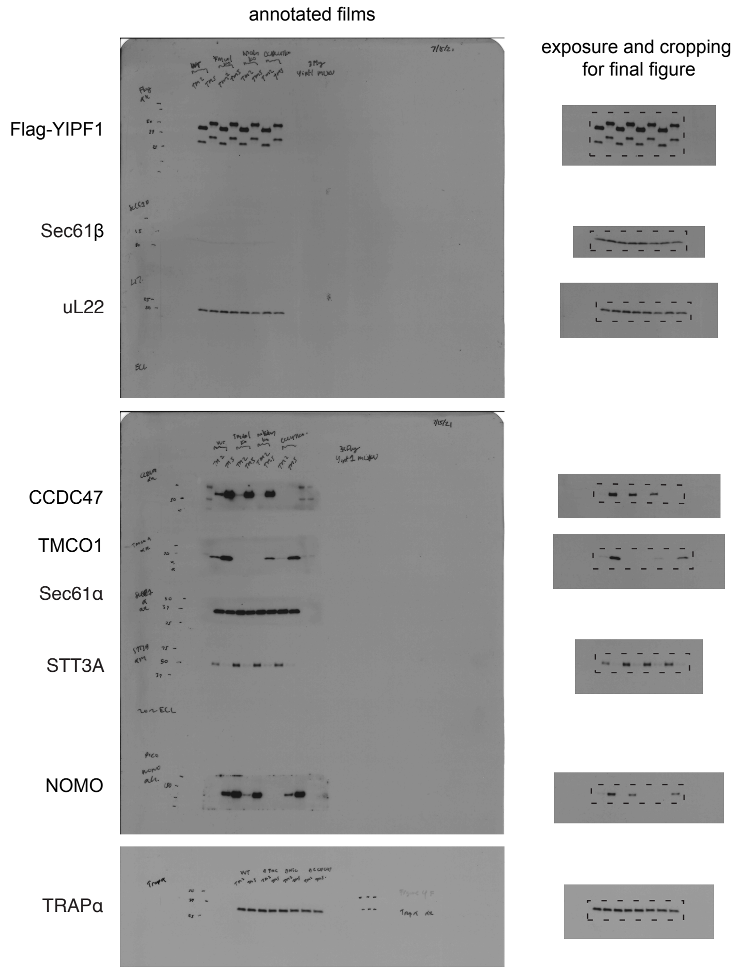

Extended Data Figure 5e

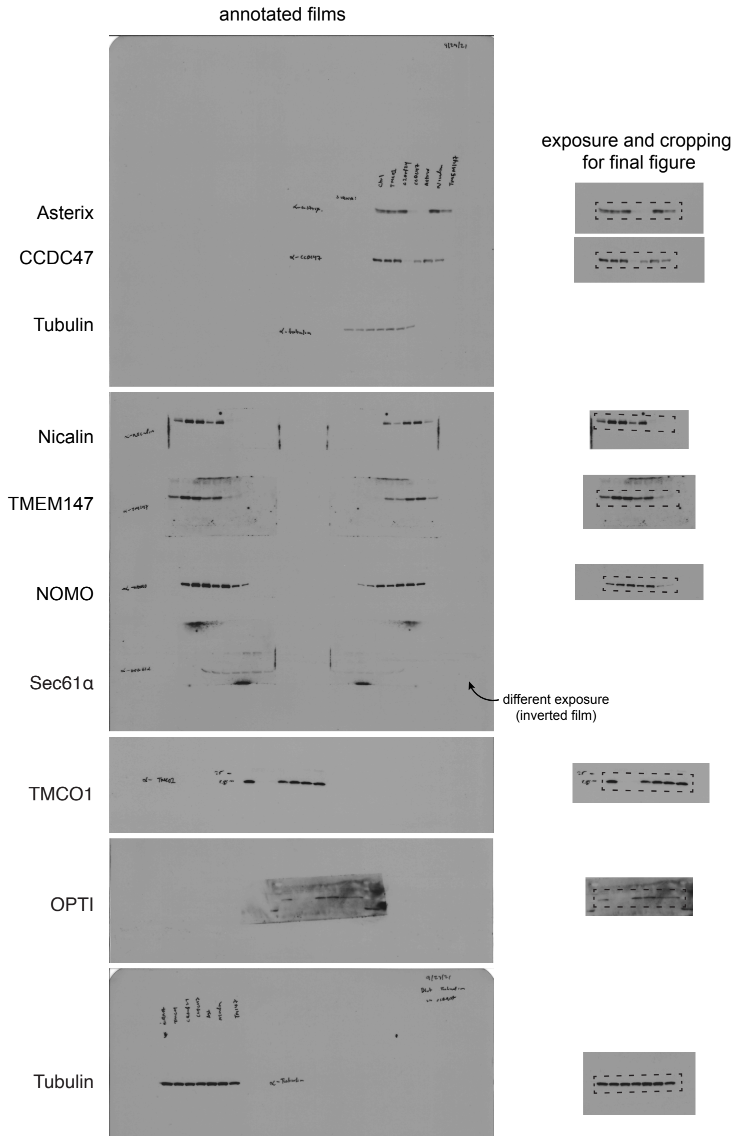

Supplementary Figure 2 l Example of gating strategy for flow cytometry analysis. Three-step gating procedure for analysis of the dual color reporter cell lines used in this study. This example is from the control siRNA-treated cell line harboring the YIPF1 reporter (Fig. 4i). The three steps are: **a**, forward and side scatter to select all cells; **b**, height versus area forward scatter to select single cells; **c**, presence of relatively high level expression of the soluble fluorescent reporter protein (either GFP or RFP, depending on the construct). Cells meeting all these criteria are plotted in the figures as histograms.

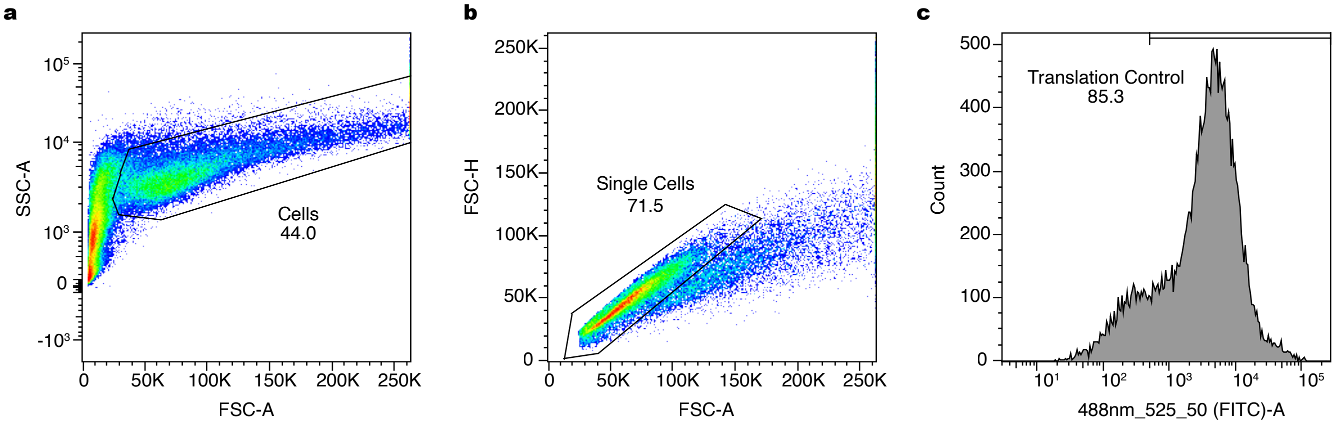

Supplement: Supplementary file 1 — This file contains Supplementary Figs. 1 and 2. [file 41586_2022_5330_MOESM1_ESM.pdf]
