# Supplementary material for: Substrate-driven assembly of a translocon for multipass membrane proteins
Source: Nature. 2022 Oct 19;611(7934):167–72. doi: 10.1038/s41586-022-05330-8 (PMC9630114; doi:10.1038/s41586-022-05330-8)
Supplement: Supplementary file 2 — Reporting Summary [file 41586_2022_5330_MOESM2_ESM.pdf]

nature portfolio

Corresponding author(s): Robert Keenan

Last updated by author(s): August 17, 2022

Reporting Summary

Nature Portfolio wishes to improve the reproducibility of the work that we publish. This form provides structure for consistency and transparency in reporting. For further information on Nature Portfolio policies, see our Editorial Policies and the Editorial Policy Checklist.

Statistics

For all statistical analyses, confirm that the following items are present in the figure legend, table legend, main text, or Methods section.

n/a	Confirmed	
☐	☒	The exact sample size (n) for each experimental group/condition, given as a discrete number and unit of measurement
☐	☒	A statement on whether measurements were taken from distinct samples or whether the same sample was measured repeatedly
☐	☒	The statistical test(s) used AND whether they are one- or two-sided<br>*Only common tests should be described solely by name; describe more complex techniques in the Methods section.*
☒	☐	A description of all covariates tested
☐	☒	A description of any assumptions or corrections, such as tests of normality and adjustment for multiple comparisons
☐	☒	A full description of the statistical parameters including central tendency (e.g. means) or other basic estimates (e.g. regression coefficient) AND variation (e.g. standard deviation) or associated estimates of uncertainty (e.g. confidence intervals)
☐	☒	For null hypothesis testing, the test statistic (e.g. F, t, r) with confidence intervals, effect sizes, degrees of freedom and P value noted<br>*Give P values as exact values whenever suitable.*
☒	☐	For Bayesian analysis, information on the choice of priors and Markov chain Monte Carlo settings
☒	☐	For hierarchical and complex designs, identification of the appropriate level for tests and full reporting of outcomes
☒	☐	Estimates of effect sizes (e.g. Cohen's d, Pearson's r), indicating how they were calculated

Our web collection on statistics for biologists contains articles on many of the points above.

Software and code

Policy information about availability of computer code

Data collection	No software was used for data collection.
Data analysis	Glycosylation was quantified using ImageJ 2.1.0/1.53n; statistical analysis was done with GraphPad Prism 9.4.0; ; flow cytometry data were analyzed using FlowJo V10.8.

For manuscripts utilizing custom algorithms or software that are central to the research but not yet described in published literature, software must be made available to editors and reviewers. We strongly encourage code deposition in a community repository (e.g. GitHub). See the Nature Portfolio guidelines for submitting code & software for further information.

Data

Policy information about availability of data

All manuscripts must include a data availability statement. This statement should provide the following information, where applicable:

- Accession codes, unique identifiers, or web links for publicly available datasets
- A description of any restrictions on data availability
- For clinical datasets or third party data, please ensure that the statement adheres to our policy

Data generated in this study are available within the article and supplementary materials. Source data for all gels can be found in Supplementary Fig. 1. The gating strategy for flow cytometry experiments can be found in Supplementary Fig.2. Source data for the graphs shown in Fig. 4e,f can be found in Supplementary Table 1. Source data for the graphs shown in Extended Data Fig. 4b can be found in Supplmentary Table 2.

Human research participants

Policy information about [studies involving human research participants and Sex and Gender in Research.](#)

Reporting on sex and gender	N/A
Population characteristics	N/A
Recruitment	N/A
Ethics oversight	N/A

Note that full information on the approval of the study protocol must also be provided in the manuscript.

Field-specific reporting

Please select the one below that is the best fit for your research. If you are not sure, read the appropriate sections before making your selection.

☒ Life sciences ☐ Behavioural & social sciences ☐ Ecological, evolutionary & environmental sciences

For a reference copy of the document with all sections, see nature.com/documents/nr-reporting-summary-flat.pdf

Life sciences study design

All studies must disclose on these points even when the disclosure is negative.

Sample size	No calculations were used to predetermine sample size. For biochemical experiments in vitro and functional assays in cells, sample sizes were determined by the magnitude of the observed effect. Typically, each experiment was repeated in part or in full at least twice. Where statistical analysis was warranted, between three and five independent biological replicates were performed. More detail is provided in the "Statistics and reproducibility" section of the Methods.
Data exclusions	No data were excluded from the analysis.
Replication	All experiments were repeated in part or in whole on separate days to verify reproducibility. All attempts at replication were successful. More detail is provided in the "Statistics and reproducibility" section of the Methods.
Randomization	Randomization is not applicable to this in vitro work as no group allocations were performed.
Blinding	Blinding is not applicable to this in vitro work as no group allocations were performed.

Reporting for specific materials, systems and methods

We require information from authors about some types of materials, experimental systems and methods used in many studies. Here, indicate whether each material, system or method listed is relevant to your study. If you are not sure if a list item applies to your research, read the appropriate section before selecting a response.

Materials & experimental systems

n/a	Involved in the study	
☐	☒	Antibodies
☐	☒	Eukaryotic cell lines
☒	☐	Palaeontology and archaeology
☒	☐	Animals and other organisms
☒	☐	Clinical data
☒	☐	Dual use research of concern

Methods

n/a	Involved in the study	
☒	☐	ChIP-seq
☐	☒	Flow cytometry
☒	☐	MRI-based neuroimaging

Antibodies

Antibodies used	Antibodies against human Sec61β (ref. 48), TRAPα (ref. 48) and TMCO1 (ref. 9) were described previously. Other antibodies were obtained from the following commercial sources: rabbit anti-Nicalin (A305-623A-M) and rabbit anti-CCDC47 (A305-100A) antibodies from Bethyl Laboratories; mouse anti-HA (326700), goat anti-NOMO (PA5-47534), rabbit anti-TMEM147 (PA5-95876), rabbit anti-Asterix (PA5-66788), rabbit anti-C20orf24 (PA5-43332), and rabbit anti-Sec61α (PA5-21773) antibodies from Invitrogen; rabbit anti-

	uL22 antibodies from Abgent (AP9892b); mouse anti-tubulin (ab11304) and mouse anti-HRP (ab6728) antibodies from Abcam; mouse anti-Flag (F1804), rabbit anti-Flag (F7425), rabbit anti-peroxidase (SAB3700863), and goat anti-peroxidase (A5420) antibodies from Sigma; mouse anti-STT3A (H00003703-M02) antibodies from Novus Biologicals; mouse anti-BiP/GRP78 (610979) antibodies from BD Biosciences.
Validation	Antibodies were validated for specificity against the human antigen by the manufacturer or in earlier published work as follows:<br><br>Anti-Sec61alpha -- validated for IB by manufacturer (Invitrogen)<br>Anti-Sec61beta -- custom antibody validated in ref. 48<br>Anti-TRAPalpha -- custom antibody validated in ref. 48<br>Anti-STT3A -- validated for IB by manufacturer (Novus)<br>Anti-Bip/GRP78 -- validated for IB by manufacturer (BD Biosciences)<br>Anti-uL22 -- validated for IB by manufacturer (Abgent)<br>Anti-TMEM147 -- validated for IB by manufacturer (Invitrogen) against mouse and in ref. 3 against human protein<br>Anti-NOMO -- validated for IB by manufacturer (Invitrogen) and in ref. 3<br>Anti-Nicalin -- validated for IB by manufacturer (Bethyl) and in ref. 3<br>Anti-CCDC47 -- validated for IB by manufacturer (Bethyl), and in refs. 3 and 15<br>Anti-Asterix -- validated for IB by manufacturer (Invitrogen) and in ref. 15<br>Anti-TMCO1 -- custom antibody validated in ref. 9<br>Anti-C20orf24/OPTI -- validated for IB by manufacturer (Invitrogen)<br>Anti-alpha-tubulin -- validated for IB by manufacturer (Abcam)<br><br>Anti-STT3A, anti-TMEM147, anti-NOMO, anti-Nicalin, anti-CCDC47, anti-Asterix, anti-TMCO1 and anti-C20orf24/OPTI were additionally validated by knockout and/or siRNA knockdown experiments as described in the manuscript.

Eukaryotic cell lines

Policy information about cell lines and Sex and Gender in Research

Cell line source(s)	Flp-In T-REx 293 Cell Line (Invitrogen)
Authentication	The Flp-In T-REx 293 cell line was authenticated by the antibiotic resistance markers within its genome.
Mycoplasma contamination	Cells were checked approximately every 6 months for mycoplasma contamination using the Universal Mycoplasma Detection Kit (ATCC), and were found to be negative
Commonly misidentified lines<br>(See ICLAC register)	None used.

Flow Cytometry

Plots

Confirm that:

☒ The axis labels state the marker and fluorochrome used (e.g. CD4-FITC).

☒ The axis scales are clearly visible. Include numbers along axes only for bottom left plot of group (a 'group' is an analysis of identical markers).

☒ All plots are contour plots with outliers or pseudocolor plots.

☒ A numerical value for number of cells or percentage (with statistics) is provided.

Methodology

Sample preparation	Cells were collected by trypsinization, washed once in ice-cold PBS, then resuspended 1 ml of PBS. Cells were passed through a 70 µm filter before flow cytometry analysis
Instrument	Becton Dickinson LSRII instrument
Software	FlowJo V10.8
Cell population abundance	No sorting was performed.
Gating strategy	Between 15,000 and 30,000 GFP-positive (EAAT1 and AGTR2) or RFP-positive (ASGR1 and YIPF1) cells were collected. Additional gating for relatively high levels of the soluble fluorescent protein reporter was used to focus on the population of cells with productive translation of reporter constructs.

☒ Tick this box to confirm that a figure exemplifying the gating strategy is provided in the Supplementary Information.
